# Sample Size Determination for Interval Estimation of the Prevalence of a Sensitive Attribute Under Randomized Response Models

**DOI:** 10.1007/s11336-022-09854-w

**Published:** 2022-03-20

**Authors:** Shi-Fang Qiu, Man-Lai Tang, Ji-Ran Tao, Ricky S. Wong

**Affiliations:** 1 Department of Statistics, Chongqing University of Technology, Chongqing, 400054 China; 2 Department of Mathematics, Brunel University London, Uxbridge, UB8 3PH UK; 3 School of Mathematics and Statistics, Beijing Institute of Technology, Beijing, 100081 China; 4 Business School, University of Hertfordshire, Hatfield, Hertfordshire, AL10 9EU UK

**Keywords:** assurance probability, confidence interval, randomized response models, sample size determination, sensitive attribute

## Abstract

Studies with sensitive questions should include a sufficient number of respondents to adequately address the research interest. While studies with an inadequate number of respondents may not yield significant conclusions, studies with an excess of respondents become wasteful of investigators’ budget. Therefore, it is an important step in survey sampling to determine the required number of participants. In this article, we derive sample size formulas based on confidence interval estimation of prevalence for four randomized response models, namely, the Warner’s randomized response model, unrelated question model, item count technique model and cheater detection model. Specifically, our sample size formulas control, with a given assurance probability, the width of a confidence interval within the planned range. Simulation results demonstrate that all formulas are accurate in terms of empirical coverage probabilities and empirical assurance probabilities. All formulas are illustrated using a real-life application about the use of unethical tactics in negotiation.

Traditional direct questioning methods face limitations when socially sensitive topics are studied. Usually, respondents may refuse to answer, may conceal their true preferences, opinions or behaviors if the attribute in question is illegal, or may temper their responses to appear to be more socially acceptable (social desirability bias), especially if their responses can be observed by the third parties. These make data collection using surveys with sensitive questions challenging, as refusal to answer will result in nonresponse bias and offering untruthful answers will lead to response bias. Both sources of bias can negatively influence data quality and produce severely biased prevalence estimates (i.e., over- or under-estimation of the behavior under study) and inflated standard error estimates (i.e., probably wrong conclusion), which in turn jeopardize the usefulness of the data for both research and policy making (Chaudhuri & Mukerjee, 1988; Rasinski et al., 1999; Tourangeau & Yan, 2007).

Randomized response techniques (RRTs), originally proposed by Warner (1965), aim to eliminate or at least minimize both response and nonresponse biases from survey respondents. By introducing random noise via employing a randomizing device, the RRT conceals individual responses and thus protects respondents’ privacy. Therefore, respondents may be more willing to answer truthfully. In Warner’s model, a respondent is presented with two mutually exclusive statements about the sensitive attributes, for example, (*A*): Have you ever made promises without an intention to deliver in a negotiation? and ($A^c$): Have you never made promises without an intention to deliver in a negotiation? The respondent is then instructed to provide an answer to statement *A* or $A^c$, depending on the outcome from a randomizing device provided by the interviewer (e.g., spinning arrow, dice or coin), with the probability *p* of being assigned to answer *A* and $1-p$ of being assigned to answer $A^c$ (with $p \ne 0.5$). It should be noted that the interviewer does not know which question to be answered by the respondent, but he or she knows only the probability *p*. As a result, the privacy of the interviewee is protected (for details, see Fox & Tracy, 1986; Horvitz, Greenberg, & Abernathy, 1976). It is noteworthy that statements A and A$^c$ are two related but mutually exclusive and complimentary questions. Most importantly, both questions are related to the sensitive attribute. Several revisions and modifications of Warner’s model have been proposed over the years (e.g., Greenberg et al., 1969; Mangat, 1994). One of these is the well-established unrelated question model (UQM) proposed by Greenberg et al. (1969). Briefly, UQM simply replaces statement $A^c$ in Warner’s model by a neutral question, for example, "Were you born in the first quarter of a year?". Dowling and Shachtman (1975) proved that UQM usually yields a more reliable sensitive prevalence estimate than Warner’s estimate. Even though an unrelated question is introduced in the UQM, some respondents ("cheaters") will choose to answer a self-protective "No" to either of the two alternative questions in the survey. To take this behavior into consideration, another modification of the RRT known as cheater detection model (CDM) was proposed by Clark and Desharnais (1998). CDM is considered as an improvement over the forced-response procedure, which modifies Warner’s model by replacing the neutral question in the UQM by the forced instruction to say "yes" for the inverted sensitive question. In particular, it considers not only those carriers of the sensitive attribute who answer honestly and noncarriers who answer honestly but also a third class of respondents, namely cheaters who respond "No" regardless of the outcome of the randomization procedure. Clark and Desharnais (1998) referred to the latter class as cheaters. To provide a greater degree of privacy to respondents, Miller (1984) developed another indirect questioning technique, namely the item count technique (ICT), in which the respondents are randomly assigned to either the experiment group or control group. Respondents in the control group are presented with a list of *k* neutral questions with answers "Yes" or "No", those in the experiment group are given the same *k* neutral questions together with the sensitive item. In both groups, all respondents are asked to report only their total number of "Yes" answers but not the replies to the individual items. Note that the response to each question is a binary variable. The difference between the observed means in the experiment and control groups provides an estimate of the proportion of the sensitive attribute. The privacy of respondents is protected to a greater extent, because this approach allows respondents to mask their answers to the sensitive question. Therefore, a potentially less biased prevalence estimate of the sensitive attribute may be obtained using the item count procedure.

Sample size determination becomes a crucial step in every study design, since studies with an inadequate number of respondents may not yield significant conclusions, while those with an excess of respondents become a waste of investigators’ resources. Ulrich et al. (2012) derived the statistical powers for the aforementioned models (i.e., Warner’s RRT model, UQM, ICT and CDM) based on the Wald test statistic. As a result, their corresponding sample size requirements that can achieve a desired power for the Wald test with a predetermined Type I error rate can be readily obtained. However, it is well documented that confidence intervals are more informative than simple hypothesis tests (which simply yield a direct accept-or-reject conclusion) in terms of description of location and precision of the statistic, and confidence intervals should be the best reporting strategy, based on the recommendations of Wilkinson and the American Psychological Association Task Force on Statistical Inference (1999) and the *Publication Manual of the American Psychological Association* (2009). Indeed, several prominent educational and psychological journals stressed in their editorial guidelines and methodological recommendations that it is necessary to include some measures of effect size and confidence intervals for all primary outcomes (see, Alhija and Levy, 2009; Dunst and Hamby, 2012; Fritz, Morris and Richler, 2012; Odgaard and Fowler, 2010; and Sun, Pan and Wang, 2010). In this paper, we thus consider the sample size formulas that can control the width of a confidence interval for the prevalence of sensitive attributes with a pre-specified confidence level. Most importantly, our formulas explicitly incorporate an assurance probability of achieving pre-specified precision.

This article is organized as follows. Sample size formulas that control the width of a confidence interval with a pre-specified confidence level for the prevalence of a sensitive attribute for the aforementioned models (i.e., Warner’s RRT model, UQM, ICT and CDM) are derived in Sect. 1. Most importantly, our formulas explicitly incorporate a pre-specified probability of achieving the pre-specified width. We evaluate their performance in Sect. 2. In Sect. 3, a real example of how Kern and Chugh (2009) examined negotiators’ unethical behavior is used to illustrate the accuracy of the estimated sample size formulas. In this example, the sensitive question is: Have you ever made promises without an intention to deliver in a negotiation? In one of Kern and Chugh’s (2009) experiments, $16.5\%$ of participants, on average, made false promises to their advantage. Suppose that an applied psychologist collects survey data and examines whether negotiators use this unethical tactic in order to increase the likelihood of reaching an agreement. We calculate the required sample size for a new study that can control the width of a confidence interval at a specified confidence level, with the assurance probability of achieving the pre-specified precision, for the various RRT models considered in this article. A brief conclusion and discussion will be given in Sect. 4.

## Sample Size Determination

### Sample Size Estimation Under Warner’s RRT Model

#### Confidence Intervals Under Warner’s RRT Model

Under Warner’s model, a randomizing device is used to determine if a respondent will answer the sensitive question A with probability *p* or the complement question A$^c$ with probability $1-p$ with *p* $\ne$ 0.5. The parameter of interest is the proportion of subjects in the population who possess the sensitive attribute and is denoted by $\pi _s$. Let $\lambda$ $=$ $\pi _s p+(1-\pi _s)(1-p)$ (i.e., the probability of a respondent answering "Yes"), *n* the number of respondents participating in the survey and *x* the number of respondents answering "Yes." Obviously, *x* follows the binomial distribution $B(n,\lambda )$ and the maximum likelihood estimate (MLE) of $\lambda$ is given by $\hat{\lambda }$ $=$ *x*/*n* with expectation being $\text{ E }(\hat{\lambda })$ $=$ $\lambda$ and variance being $\text{ Var }(\hat{\lambda })$ $=$ $\lambda (1-\lambda )/n$. Since $\pi _s$ $=$ $(\lambda +p-1)/(2p-1)$ (*p* $\ne$ 0.5), the MLE of $\pi _s$ is given by $\hat{\pi }_{s, WM}$ $=$ $(\hat{\lambda }+p-1)/(2p-1)$. Therefore, the variance of $\hat{\pi }_s$ is given by

1 $$\begin{aligned} \text{ Var }(\hat{\pi }_{s, WM})=\frac{\lambda (1-\lambda )}{n(2p-1)^2}. \end{aligned}$$

As a result, the $(1-\alpha )100\%$ Wald confidence interval (CI) for $\pi _s$ is given by

2 $$\begin{aligned} \text{ CI}_{{WM, W}}=\left[ \hat{\pi }_{s, WM}-z_{\alpha /2}\sqrt{\frac{\hat{\lambda }(1-\hat{\lambda })}{n(2p-1)^2}}, \hat{\pi }_{s, WM}+z_{\alpha /2}\sqrt{\frac{\hat{\lambda }(1-\hat{\lambda })}{n(2p-1)^2}}\right] , \end{aligned}$$

where $z_{\alpha /2}$ is the $1 -\alpha /2$ quantile of the standard normal distribution. As shown in Newcombe (1998) and Agresti and Coull (1998), a confidence interval based on Wilson method performs very well compared to the Wald interval, when sample size is not large. Therefore, we also apply the Wilson (1927) method to construct a $(1-\alpha )100\%$ confidence interval for $\pi _s$ as

3 $$\begin{aligned} \text{ CI}_{WM, Wi}=[\pi _{l, WM, Wi},\pi _{u, WM, Wi}], \end{aligned}$$

where

$$\begin{aligned}&\pi _{l, WM, Wi} =\frac{n\hat{\lambda }+z_{\alpha /2}^2/2+(p-1)(n+z_{\alpha /2}^2)-z_{\alpha /2}\sqrt{n\hat{\lambda }(1-\hat{\lambda })+z_{\alpha /2}^2/4}}{|2p-1|(n+z_{\alpha /2}^2)} \text{ and } \\&\pi _{u, WM, Wi}=\frac{n\hat{\lambda }+z_{\alpha /2}^2/2+(p-1)(n+z_{\alpha /2}^2)+z_{\alpha /2}\sqrt{n\hat{\lambda }(1-\hat{\lambda })+z_{\alpha /2}^2/4}}{|2p-1|(n+z_{\alpha /2}^2)}. \end{aligned}$$

#### Sample Size Formula Based on Wald Confidence Interval ${{\varvec{[n_{WM, W}}}}$, ${{\varvec{n_{WM, W, 0.5}]}}}$

The half width of the $(1-\alpha )100\%$ Wald CI for $\pi _s$ is given by

$$\begin{aligned} z_{\alpha /2}\sqrt{\frac{\hat{\lambda }(1-\hat{\lambda })}{n(2p-1)^2}}. \end{aligned}$$

Here, we control the half width no larger than $\omega$ with probability $1-\beta$. That is,

$$\begin{aligned} Pr(z_{\alpha /2}\sqrt{\frac{\hat{\lambda }(1-\hat{\lambda })}{n(2p-1)^2}}\le \omega )\ge 1-\beta , \end{aligned}$$

or equivalently

$$\begin{aligned} Pr(\sqrt{\hat{\lambda }(1-\hat{\lambda })}\le \frac{\omega |2p-1|\sqrt{n}}{z_{\alpha /2}})\ge 1-\beta . \end{aligned}$$

According to the delta method, it is easily shown that

$$\begin{aligned} \sqrt{\hat{\lambda }(1-\hat{\lambda })}\sim N(\sqrt{\lambda (1-\lambda )},\frac{(1-2\lambda )^2}{4n}). \end{aligned}$$

If we let *Z* $=$ $\frac{\sqrt{\hat{\lambda }(1-\hat{\lambda })}-\sqrt{\lambda (1-\lambda )}}{|1-2\lambda |/(2\sqrt{n})}$, then we have

$$\begin{aligned} Pr(Z\le \frac{\frac{\omega |2p-1|\sqrt{n}}{z_{\alpha /2}}-\sqrt{\lambda (1-\lambda )}}{|1-2\lambda |/(2\sqrt{n})})_\ge 1-\beta . \end{aligned}$$

Therefore, the desired sample size *n* satisfies the following equation:

$$\begin{aligned} \frac{\omega |2p-1|\sqrt{n}}{z_{\alpha /2}}-\sqrt{\lambda (1-\lambda )}=z_\beta |1-2\lambda |/(2\sqrt{n}), \end{aligned}$$

where $z_\beta$ is the $1-\beta$ quantile of a standard normal distribution.

Solving the above equation yields

4 $$\begin{aligned} {{\varvec{n_{WM, W}}}}=\Big [\frac{[\lambda (1-\lambda )]^{1/2}+[\lambda (1-\lambda )+2\omega |2p-1||1-2\lambda |z_\beta /z_{\alpha /2}]^{1/2}}{2\omega |2p-1|/z_{\alpha /2}}\Big ]^2. \end{aligned}$$

In particular, when $\beta$ $=$ 0.5 the conventional sample size is given by

5 $$\begin{aligned} {{\varvec{n_{WM, W, 0.5}}}} =\frac{\lambda (1-\lambda )}{[\omega (2p-1)/z_{\alpha /2}]^2}. \end{aligned}$$

Given the values of *n*, *p* and $\pi _s$, the assurance probability can be obtained by

$$\begin{aligned} \Phi \left( \frac{\frac{\omega |2p-1|\sqrt{n}}{z_{\alpha /2}}-\sqrt{\lambda (1-\lambda )}}{|1-2\lambda |/(2\sqrt{n})}\right) , \end{aligned}$$

where $\Phi (\cdot )$ is the distribution function of the standard normal random variable.

#### Sample Size Formula Based on Wilson Confidence Interval ${{\varvec{[n_{WM, Wi}}}}$, ${{\varvec{n_{WM, Wi, 0.5}]}}}$

Similarly, in order to control the half width of the Wilson CI to be no larger than $\omega$ with probability $1-\beta$, the sample size estimate needs to satisfy

$$\begin{aligned} Pr\left( \frac{z_{\alpha /2}\sqrt{n\hat{\lambda }(1-\hat{\lambda })+z_{\alpha /2}^2/4}}{|2p-1|(n+z_{\alpha /2}^2)}\le \omega \right) \ge 1-\beta , \end{aligned}$$

i.e.,

$$\begin{aligned} Pr\left( \hat{\lambda }(1-\hat{\lambda })\le \frac{4(2p-1)^2(n+z_{\alpha /2}^2)^2\omega ^2-z_{\alpha /2}^4}{4nz_{\alpha /2}^2}\right) \ge 1-\beta . \end{aligned}$$

According to the delta method, the asymptotic distribution of $\hat{\lambda }(1-\hat{\lambda })$ is

$$\begin{aligned} \hat{\lambda }(1-\hat{\lambda })\sim N(\lambda (1-\lambda ),\lambda (1-\lambda )(1-2\lambda )^2/n). \end{aligned}$$

Therefore,

$$\begin{aligned} \frac{4(2p-1)^2(n+z_{\alpha /2}^2)^2\omega ^2-z_{\alpha /2}^4}{4nz_{\alpha /2}^2}-\lambda (1-\lambda )=z_{\beta }\sqrt{\lambda (1-\lambda )(1-2\lambda )^2/n}, \end{aligned}$$

i.e., the approximate sample size (denoted as ${{\varvec{n_{WM, Wi}}}}$) can be obtained by solving the following equation:

6 $$\begin{aligned} a(n+z_{\alpha /2}^2)^4+b(n+z_{\alpha /2}^2)^3+c(n+z_{\alpha /2}^2)^2+d(n+z_{\alpha /2}^2)+e=0, \end{aligned}$$

where

$$\begin{aligned} a= & {} 16\omega ^4(2p-1)^4,\\ b= & {} -32z_{\alpha /2}^2\omega ^2(2p-1)^2\lambda (1-\lambda ),\\ c= & {} 8z_{\alpha /2}^4[2\lambda ^2(1-\lambda )^2+4\omega ^2(2p-1)^2\lambda (1-\lambda )-\omega ^2(2p-1)^2],\\ d= & {} 8z_{\alpha /2}^4[z_{\alpha /2}^2\lambda (1-\lambda )-4z_{\alpha /2}^2\lambda ^2(1-\lambda )^2-2z_\beta ^2\lambda (1-\lambda )(1-2\lambda )^2], \text{ and }\\ e= & {} z_{\alpha /2}^6[16z_{\alpha /2}^2\lambda ^2(1-\lambda )^2+16z_\beta ^2\lambda (1-\lambda )(1-2\lambda )^2-8z_{\alpha /2}^2\lambda (1-\lambda )+z_{\alpha /2}^2]. \end{aligned}$$

The eigenvalue methods for finding roots of polynomials can be used to obtain the sample size estimate, based on Wilson method by solving Eq. (6) with respect to $n+z_{\alpha /2}^2$. If $m_\mathrm{max}$ is the maximum real root of Eq. (6), then the desired sample size ${{\varvec{n_{WM, Wi}}}}$ is the minimum integer that is not smaller than $m_\mathrm{max}-z_{\alpha /2}^2$.

In particular, when $\beta$ $=$ 0.5, the approximate sample size n is given by

7 $$\begin{aligned} {{\varvec{n_{WM, Wi, 0.5}}}} =\frac{z_{\alpha /2}^2[\lambda (1-\lambda )+\sqrt{\lambda ^2(1-\lambda )^2 +\omega ^2(2p-1)^2(1-4\lambda (1-\lambda ))}]}{2\omega ^2(2p-1)^2}-z_{\alpha /2}^2. \end{aligned}$$

Given the values of *n*, *p* and $\pi _s$, the assurance probability can be obtained by

$$\begin{aligned} \Phi \left( \frac{\frac{4(2p-1)^2(n+z_{\alpha /2}^2)^2\omega ^2-z_{\alpha /2}^4}{4nz_{\alpha /2}^2}-\lambda (1-\lambda )}{\sqrt{\lambda (1-\lambda )(1-2\lambda )^2/n}}\right) . \end{aligned}$$

### Sample Size Estimation Under Unrelated Question Model

#### Confidence Intervals Under Unrelated Question Model

It should be noted that the unrelated question model (UQM) simply replaces question A$^c$ in Warner’s model with a neutral question N (see, Greenberg et al., 1969). Similar to Warner’s model, a randomizing device is used to impel a respondent to answer A with a probability of *p* or the neutral question N with a probability of $1 - p$. Let the probability of responding "yes" to statement N be $\pi _N$. If $\lambda$ $=$ $p\pi _s+(1-p)\pi _N$, then *x* follows binomial distribution B(n,$\lambda$). Again, the MLE of $\lambda$ is given by $\hat{\lambda }$ $=$ *x*/*n*, and the expectation and variance of $\hat{\lambda }$ are, respectively, E($\hat{\lambda }$) $=$ $\lambda$ and Var($\hat{\lambda }$) $=$ $\lambda (1-\lambda )/n$. Therefore, the MLE of $\pi _{s, UQM}$ is $\hat{\pi }_{s, UQM}$ $=$ $(\hat{\lambda }-(1-p)\pi _N)/p$, E($\hat{\pi }_{s, UQM}$) $=$ $\pi _s$ and Var($\hat{\pi }_{s, UQM}$) $=$ $\lambda (1-\lambda )/(np^2)$.

The $(1-\alpha )100\%$ confidence interval for $\pi _s$ based on Wald method is given by

8 $$\begin{aligned} \text{ CI}_{UQM, W}=[\hat{\pi }_{UQM, W}-z_{\alpha /2}\sqrt{\hat{\lambda }(1-\hat{\lambda })/(np^2)},\hat{\pi }_{UQM, W} +z_{\alpha /2}\sqrt{\hat{\lambda }(1-\hat{\lambda })/(np^2)}]. \end{aligned}$$

Alternatively, a $(1-\alpha )100\%$ Wilson confidence interval for $\lambda$ is given by

$$\begin{aligned} \frac{2n\hat{\lambda }+z_{\alpha /2}^2\mp \sqrt{z_{\alpha /2}^4+4nz_{\alpha /2}^2\hat{\lambda }(1-\hat{\lambda })}}{2(n+z_{\alpha /2}^2)}. \end{aligned}$$

Hence, the $(1-\alpha )100\%$ Wilson confidence interval for $\pi _s$ can be obtained as

9 $$\begin{aligned} \text{ CI}_{UQM, Wi}=[\pi _{l, UQM, Wi},\pi _{u, UQM, Wi}], \end{aligned}$$

where

$$\begin{aligned}&\pi _{l, UQM, Wi}\\&\quad =\frac{n\hat{\lambda }+z_{\alpha /2}^2/2-(1-p)(n+z_{\alpha /2}^2)\pi _N- z_{\alpha /2}\sqrt{z_{\alpha /2}^2/4+n\hat{\lambda }(1-\hat{\lambda })}}{p(n+z_{\alpha /2}^2)} \end{aligned}$$

and

$$\begin{aligned}&\pi _{u, UQM, Wi}\\&\quad =\frac{n\hat{\lambda }+z_{\alpha /2}^2/2-(1-p)(n+z_{\alpha /2}^2)\pi _N+ z_{\alpha /2}\sqrt{z_{\alpha /2}^2/4+n\hat{\lambda }(1-\hat{\lambda })}}{p(n+z_{\alpha /2}^2)}. \end{aligned}$$

#### Sample Size Formula Based on Wald Confidence Interval ${{\varvec{[n_{UQM, W}}}}$, ${{\varvec{n_{UQM, W, 0.5}]}}}$

Again, we control the half width of the Wald CI to be no larger than $\omega$ with probability $1-\beta$. That is,

$$\begin{aligned} Pr(z_{\alpha /2}\sqrt{\hat{\lambda }(1-\hat{\lambda })/(np^2)}\le \omega )\ge 1-\beta , \end{aligned}$$

or,

10 $$\begin{aligned} Pr(\sqrt{\hat{\lambda }(1-\hat{\lambda })}\le \omega p\sqrt{n}/z_{\alpha /2})\ge 1-\beta . \end{aligned}$$

It is shown that

$$\begin{aligned} \sqrt{\hat{\lambda }(1-\hat{\lambda })}\sim N(\sqrt{\lambda (1-\lambda )},(1-2\lambda )^2/(4n)). \end{aligned}$$

Equation (10) becomes

$$\begin{aligned} Pr\left( \frac{\sqrt{\hat{\lambda }(1-\hat{\lambda })}-\sqrt{\lambda (1-\lambda )}}{|1-2\lambda |/(2\sqrt{n})}\le \frac{\omega p\sqrt{n}/z_{\alpha /2}-\sqrt{\lambda (1-\lambda )}}{|1-2\lambda |/(2\sqrt{n})}\right) \ge 1-\beta . \end{aligned}$$

Therefore,

$$\begin{aligned} \frac{\omega p\sqrt{n}/z_{\alpha /2}-\sqrt{\lambda (1-\lambda )}}{|1-2\lambda |/(2\sqrt{n})}=z_{\beta }, \end{aligned}$$

i.e.,

$$\begin{aligned} \frac{2\omega p}{z_{\alpha /2}}(\sqrt{n})^2-2\sqrt{\lambda (1-\lambda )}\cdot \sqrt{n}-z_{\beta }|1-2\lambda |=0. \end{aligned}$$

By solving the above equation, we have

11 $$\begin{aligned} {{\varvec{n_{UQM, W}}}}=\Big [\frac{z_{\alpha /2}\sqrt{\lambda (1-\lambda )}+\sqrt{z_{\alpha /2}^2\lambda (1-\lambda )+ 2z_{\alpha /2}\omega pz_\beta |1-2\lambda |}}{2\omega p}\Big ]^2. \end{aligned}$$

When $\beta$ $=$ 0.5, the approximate sample size is given by

12 $$\begin{aligned} {{\varvec{n_{UQM, W, 0.5}}}}=\lambda (1-\lambda )/(\omega p/z_{\alpha /2})^2. \end{aligned}$$

#### Sample Size Formula Based on Wilson Confidence Interval ${{\varvec{[n_{UQM, Wi}}}}$, ${{\varvec{n_{UQM, Wi, 0.5}]}}}$

To control the half width of the Wilson CI that is no larger than $\omega$ with probability $1-\beta$, the desired sample size should satisfy

$$\begin{aligned} Pr\left( z_{\alpha /2}\sqrt{n\hat{\lambda }(1-\hat{\lambda })+z_{\alpha /2}^2/4}/ \left[ p(n+z_{\alpha /2}^2)\right] \le \omega \right) \ge 1-\beta , \end{aligned}$$

i.e.,

13 $$\begin{aligned} Pr\left( \hat{\lambda }(1-\hat{\lambda })\le \frac{4\omega ^2p^2(n+z_{\alpha /2}^2)-z_{\alpha /2}^4}{4nz_{\alpha /2}^2}\right) \ge 1-\beta . \end{aligned}$$

It is thus shown that the sample size is the solution to the following equation:

14 $$\begin{aligned} a(n+z_{\alpha /2}^2)^4+b(n+z_{\alpha /2}^2)^3+c(n+z_{\alpha /2}^2)^2+d(n+z_{\alpha /2}^2)+e=0, \end{aligned}$$

where

$$\begin{aligned} \begin{array}{lll} a&{}=16p^4\omega ^4,\\ b&{}=-32p^2\omega ^2z_{\alpha /2}^2\lambda (1-\lambda ),\\ c&{}=8z_{\alpha /2}^4\left[ 2\lambda ^2(1-\lambda )^2+4p^2\omega ^2\lambda (1-\lambda )-p^2\omega ^2\right] ,\\ d&{}=8z_{\alpha /2}^4\left[ z_{\alpha /2}^2\lambda (1-\lambda )-4z_{\alpha /2}^2\lambda ^2(1-\lambda )^2-2z_\beta ^2\lambda (1-\lambda )(1-2\lambda )^2\right] , \text{ and }\\ e&{}=z_{\alpha /2}^6\left[ 16z_{\alpha /2}^2\lambda ^2(1-\lambda )^2+16z_\beta ^2\lambda (1-\lambda )(1-2\lambda )^2+z_{\alpha /2}^2 -8z_{\alpha /2}^2\lambda (1-\lambda )\right] . \end{array} \end{aligned}$$

Similarly, the eigenvalue methods can be used to find the solutions of the above equation with respect to $n+z_{\alpha /2}^2$, and the approximate sample size *n* is denoted as ${{\varvec{n_{UQM, Wi}}}}$.

When $\beta$ $=$ 0.5, the formula reduces to

15 $$\begin{aligned} {{\varvec{n_{UQM,Wi,0.5}}}}=\frac{z_{\alpha /2}^2\Big [\lambda (1-\lambda )+\sqrt{\lambda ^2(1-\lambda )^2+\omega ^2p^2[1-4\lambda (1-\lambda )]}-2\omega ^2p^2\Big ]}{2\omega ^2p^2}. \end{aligned}$$

### Sample Size Estimation Under Item Count Technique

#### Confidence Intervals Under Item Count Technique

Under the item count technique (ICT) model developed by Miller (1984), it was designed that the $n_c$ respondents randomly assigned to the control group would receive a list of *k* neutral questions, while the $n_e$ respondents randomly assigned to the experiment group would receive the same set of neutral questions as the control group together with the sensitive question. The probability of answering the *i*th neutral question with "yes" would be $\pi _i$ ($i=1,2,\ldots ,k$). Let the expected numbers of total "yes" response of the control and experiment groups are $T_c$ $=$ $\sum \nolimits _{i=1}^k\pi _i$ and $T_e$ $=$ $\sum \nolimits _{i=1}^k\pi _i+\pi _s$, respectively. Let $t_c$ be the total number of answering "Yes" in the control group and $t_e$ be the total number of answering "Yes" in the experiment group, respectively. Therefore, $\hat{T}_c$ $=$ $t_c/n_c$ and $\hat{T}_e$ $=$ $t_e/n_e$, and the estimation of $\pi _s$ is given by $\hat{\pi }_s$ $=$ $\hat{T}_e$ − $\hat{T}_c$ $=$ $t_e/n_e-t_c/n_c$.

Given that all items are statistically unrelated, it is shown that the sample variance of $\hat{\pi }_s$ is given by

$$\begin{aligned} \text{ Var }(\hat{\pi }_s)=\frac{\pi _s(1-\pi _s)}{n_e}+\left( \frac{1}{n_e}+\frac{1}{n_c}\right) \sum \limits _{i=1}^k\pi _i(1-\pi _i). \end{aligned}$$

Therefore, the $(1-\alpha )100\%$ Wald confidence interval for $\pi _s$ is given by

16 $$\begin{aligned} \text{ CI}_{ICT, W}=[\pi _{l, ICT, W},\pi _{u, ICT, W}], \end{aligned}$$

where

$$\begin{aligned} \pi _{l, ICT, W}=\hat{\pi }_s-z_{\alpha /2}\left[ \frac{\hat{\pi }_s(1-\hat{\pi }_s)}{n_e}+\left( \frac{1}{n_c}+\frac{1}{n_e}\right) \sum \limits _{i=1}^k\pi _i(1-\pi _i)\right] ^{1/2} \end{aligned}$$

and

$$\begin{aligned} \pi _{u, ICT, W} =\hat{\pi }_s+z_{\alpha /2}\left[ \frac{\hat{\pi }_s(1-\hat{\pi }_s)}{n_e}+\left( \frac{1}{n_c}+\frac{1}{n_e}\right) \sum \limits _{i=1}^k\pi _i(1-\pi _i)\right] ^{1/2}, \end{aligned}$$

while the $(1-\alpha )100\%$ Wilson confidence interval for $\pi _s$ is given by

17 $$\begin{aligned} \text{ CI}_{ICT, Wi}=[\pi _{l, ICT, Wi},\pi _{u, ICT, Wi}], \end{aligned}$$

where

$$\begin{aligned}&\pi _{l, ICT, Wi}\\&\quad =\frac{n_e\hat{\pi }_s+z_{\alpha /2}^2/2-z_{\alpha /2}\left[ n_e\hat{\pi }_s(1-\hat{\pi }_s)+\left( 1+\frac{n_e}{n_c}\right) \left( n_e+z_{\alpha /2}^2\right) \sum \nolimits _{i=1}^k\pi _i(1-\pi _i)+z_{\alpha /2}^2/4\right] ^{1/2}}{n_e+z_{\alpha /2}^2} \end{aligned}$$

and

$$\begin{aligned}&\pi _{u, ICT, Wi}\\&\quad =\frac{n_e\hat{\pi }_s+z_{\alpha /2}^2/2+z_{\alpha /2}\left[ n_e\hat{\pi }_s(1-\hat{\pi }_s)+\left( 1+\frac{n_e}{n_c}\right) \left( n_e+z_{\alpha /2}^2\right) \sum \nolimits _{i=1}^k\pi _i(1-\pi _i)+z_{\alpha /2}^2/4\right] ^{1/2}}{n_e+z_{\alpha /2}^2}. \end{aligned}$$

#### Sample Size Formula Based on Wald Confidence Interval ${{\varvec{[n_{ICT, W}}}}$, ${{\varvec{n_{ICT, W, 0.5}]}}}$

It is noted that the half width of the $(1-\alpha )100\%$ Wald CI is given by

$$\begin{aligned} z_{\alpha /2}\left[ \frac{\hat{\pi }_s(1-\hat{\pi }_s)}{n_e}+\left( \frac{1}{n_e}+\frac{1}{n_c}\right) \sum \limits _{i=1}^k\pi _i(1-\pi _i)\right] ^{1/2}. \end{aligned}$$

To control the half width of the Wald CI that is no larger than $\omega$ with a probability of $1 - \beta$, the desired sample size should satisfy

$$\begin{aligned} Pr\left( z_{\alpha /2}\left[ \frac{\hat{\pi }_s(1-\hat{\pi }_s)}{n_e}+\left( \frac{1}{n_e}+\frac{1}{n_c}\right) \sum \limits _{i=1}^k\pi _i(1-\pi _i)\right] ^{1/2} \le \omega \right) \ge 1-\beta , \end{aligned}$$

i.e.,

$$\begin{aligned} Pr\left( \hat{\pi }_s(1-\hat{\pi }_s)\le \frac{n_e\omega ^2}{z_{\alpha /2}^2}-\left( 1+\frac{n_e}{n_c}\right) \sum \limits _{i=1}^k\pi _i(1-\pi _i)\right) \ge 1-\beta . \end{aligned}$$

It is clear that

$$\begin{aligned} \hat{\pi }_s(1-\hat{\pi }_s)\sim N\left( \pi _s(1-\pi _s),(1-2\pi _s)^2\left( \frac{\pi _s(1-\pi _s)}{n_e}+\left( \frac{1}{n_e}+\frac{1}{n_c}\right) \sum \limits _{i=1}^k\pi _i(1-\pi _i)\right) \right) . \end{aligned}$$

Hence, we have

$$\begin{aligned} \frac{\frac{n_e\omega ^2}{z_{\alpha /2}^2}-(1+\frac{n_e}{n_c})\sum \limits _{i=1}^k\pi _i(1-\pi _i)-\pi _s(1-\pi _s)}{|1-2\pi _s| [\frac{\pi _s(1-\pi _s)}{n_e}+(\frac{1}{n_e}+\frac{1}{n_c})\sum \limits _{i=1}^k\pi _i(1-\pi _i)]^{1/2}}=z_\beta . \end{aligned}$$

When $n_c$ $=$ $n_e$ $=$ $\frac{1}{2}n$ and let *c* $=$ $2\sum \nolimits _{i=1}^k\pi _i(1-\pi _i)+\pi _s(1-\pi _s)$, the above equation can be simplified to

18 $$\begin{aligned} n_e^3-b_1n_e^2+b_2n_e-b_3=0, \end{aligned}$$

where

$$\begin{aligned} b_1=\frac{2cz_{\alpha /2}^2}{\omega ^2},~b_2=\frac{c^2z_{\alpha /2}^4}{\omega ^4},~\mathrm{and}~b_3= \frac{(1-2\pi _s)^2cz_\beta ^2z_{\alpha /2}^4}{\omega ^4}. \end{aligned}$$

According to Cardans formula for solving the cubic equation, the desired sample size $n_e$ ($=\frac{1}{2}n$) can be estimated to be the unique real root or the maximum root of three real roots. Therefore, the estimated sample size is given by ${{\varvec{n_{ICT, W}}}}=2n_e$.

When $\beta$ $=$ 0.5, the estimated sample size *n* is given by

19 $$\begin{aligned} {{\varvec{n_{ICT, W, 0.5}}}}=2cz_{\alpha /2}^2/\omega ^2. \end{aligned}$$

#### Sample Size Formula Based on Wilson Confidence Interval ${{\varvec{[n_{ICT, Wi}}}}$, ${{\varvec{n_{ICT, Wi, 0.5}]}}}$

The half width of the $(1-\alpha )100\%$ Wilson CI is given by

$$\begin{aligned} \frac{z_{\alpha /2}\left[ z_{\alpha /2}^2/4+n_e\hat{\pi }_s(1-\hat{\pi }_s)+(1+\frac{n_e}{n_c})(n_e+z_{\alpha /2}^2)\sum \nolimits _{i=1}^k\pi _i(1-\pi _i)\right] ^{1/2}}{n_e+z_{\alpha /2}^2}. \end{aligned}$$

To control the half width of the Wilson CI to be no larger than $\omega$ with probability $1-\beta$, we have

$$\begin{aligned} Pr\left( \frac{z_{\alpha /2}\left[ z_{\alpha /2}^2/4+n_e\hat{\pi }_s(1-\hat{\pi }_s)+(1+\frac{n_e}{n_c})(n_e+z_{\alpha /2}^2)\sum \nolimits _{i=1}^k\pi _i(1-\pi _i)\right] ^{1/2}}{n_e+z_{\alpha /2}^2}\le \omega \right) \ge 1-\beta , \end{aligned}$$

i.e.,

$$\begin{aligned}&Pr(\hat{\pi }_s(1-\hat{\pi }_s)\\&\quad \le \frac{\omega ^2(n_e+z_{\alpha /2}^2)^2/z_{\alpha /2}^2-z_{\alpha /2}^2/4-(1+\frac{n_e}{n_c}) (n_e+z_{\alpha /2}^2)\sum \nolimits _{i=1}^k\pi _i(1-\pi _i)}{n_e})\ge 1-\beta . \end{aligned}$$

Similarly, it is clear that

$$\begin{aligned} \hat{\pi }_s(1-\hat{\pi }_s)\sim N(\pi _s(1-\pi _s),(1-2\pi _s)^2\left[ \frac{\pi _s(1-\pi _s)}{n_e}+\left( \frac{1}{n_e}+\frac{1}{n_c}\right) \sum \limits _{i=1}^k\pi _i(1-\pi _i)\right] ) \end{aligned}$$

Therefore, we have

$$\begin{aligned} \frac{\frac{\omega ^2(n_e+z_{\alpha /2}^2)^2/z_{\alpha /2}^2-z_{\alpha /2}^2/4-(1+\frac{n_e}{n_c}) (n_e+z_{\alpha /2}^2)\sum \limits _{i=1}^k\pi _i(1-\pi _i)}{n_e}-\pi _s(1-\pi _s)}{|1-2\pi _s| [\frac{\pi _s(1-\pi _s)}{n_e}+(\frac{1}{n_e}+\frac{1}{n_c})\sum \limits _{i=1}^k\pi _i(1-\pi _i)]^{1/2}}=z_\beta . \end{aligned}$$

If $$n_c$$ $$=$$ $$n_e$$, then

20 $$\begin{aligned} a(n_e+z_{\alpha /2}^2)^4+b(n_e+z_{\alpha /2}^2)^3+c(n_e+z_{\alpha /2}^2)^2+d(n_e+z_{\alpha /2}^2)+e=0, \end{aligned}$$

where

$$\begin{aligned} \begin{array}{lll} a=\omega ^4/z_{\alpha /2}^4,\\ b=-2\omega ^2/z_{\alpha /2}^2\left( 2\sum \limits _{i=1}^k\pi _i(1-\pi _i)+\pi _s(1-\pi _s)\right) ,\\ c=2\omega ^2\left[ -1/4+\pi _s(1-\pi _s)\right] +\left[ 2\sum \limits _{i=1}^k\pi _i(1-\pi _i)+\pi _s(1-\pi _s)\right] ^2,\\ d=-\left[ z_{\alpha /2}^2(-1/2+2\pi _s(1-\pi _s))+z_\beta ^2(1-2\pi _s)^2\right] \left[ 2\sum \limits _{i=1}^k\pi _i(1-\pi _i)+\pi _s(1-\pi _s)\right] , \text{ and }\\ e=z_{\alpha /2}^4\left[ -1/4+\pi _s(1-\pi _s)\right] ^2+z_\beta ^2(1-2\pi _s)^2z_{\alpha /2}^2\left( 2\sum \limits _{i=1}^k\pi _i(1-\pi _i)+\pi _s(1-\pi _s)\right) . \end{array} \end{aligned}$$

Similarly, the eigenvalue methods can be employed to find the solutions of the above equation with respect to $n_e+z_{\alpha /2}^2$, and the desired sample size is denoted as ${{\varvec{n_{ICT, Wi}}}}$ (i.e., ${{\varvec{n_{ICT, Wi}}}}= 2n_e$).

When $\beta$ $=$ 0.5, the estimated sample size *n* is given by

21 $$\begin{aligned} {{\varvec{n_{ICT, Wi, 0.5}}}}=\frac{z_{\alpha /2}^2(f+\sqrt{f^2-g}-\omega ^2)}{\omega ^2}, \end{aligned}$$

where *f* $$=$$ $$2\sum \nolimits _{i=1}^k\pi _i(1-\pi _i)+\pi _s(1-\pi _s)$$ and *g* $$=$$ $$\omega ^2[4\pi _s(1-\pi _s)-1]$$.

### Sample Size Estimation Under Cheater Detection Model

#### Confidence intervals Under Cheater Detection Model

Assume that the true proportions of honest-yes respondents, honest-no respondents, and cheaters are $\pi _s$, $\beta _s$ and $\gamma$, respectively (i.e., $\pi _s$+$\beta _s$+$\gamma$=1), and a total of *n* individuals participates in the interview under Cheater Detection Model (CDM). Following Clark & Desharnais (1998), the whole sample *n* is divided into two subsamples of sizes $n_1$ and $n_2$ to estimate parameters $\pi _s$, $\beta _s$ and $\gamma$. The probabilities of a respondent being assigned by the randomizing device to answer the sensitive question are, respectively, $p_1$ and $p_2$ ($p_1$ $\ne$ $p_2$) for subsamples 1 and 2. Let $\lambda _1$ and $\lambda _2$ denote the true proportions of "yes" responses, and $y_1$ and $y_2$ the number of "Yes" answers for subsamples 1 and 2. Obviously, $y_i$ follows B($n_i,\lambda _i$) (*i* =1, 2). Then, we have $\hat{\lambda }_i$ $=$ $y_i/n_i$ (*i* =1, 2), $\hat{\pi }_s$ $=$ $(p_2\hat{\lambda }_1-p_1\hat{\lambda }_2)/(p_2-p_1)$, $\hat{\beta }_s$ $=$ $(\hat{\lambda }_2-\hat{\lambda }_1)/(p_2-p_1)$ and $\hat{\gamma }$ $=$ $1-\hat{\pi }_s-\hat{\beta }_s$. Therefore, the variance of $\hat{\pi }_s$ is given by $\frac{p_2^2}{(p_2-p_1)^2}\cdot \frac{\lambda _1(1-\lambda _1)}{n_1}$ + $\frac{p_1^2}{(p_2-p_1)^2}\cdot \frac{\lambda _2(1-\lambda _2)}{n_2}$. As a result, the $(1-\alpha )100\%$ Wald confidence interval for $\pi _s$ is given by

22 $$\begin{aligned} \text{ CI}_{CDM,W}=[\pi _{l, CDM, W},\pi _{u, CDM, W}], \end{aligned}$$

where

$$\begin{aligned} \pi _{l, CDM, W} = \hat{\pi }_s-z_{\alpha /2}\left[ \frac{p_2^2}{(p_2-p_1)^2}\cdot \frac{\hat{\lambda }_1(1-\hat{\lambda }_1)}{n_1} +\frac{p_1^2}{(p_2-p_1)^2}\cdot \frac{\hat{\lambda }_2(1-\hat{\lambda }_2)}{n_2}\right] ^{1/2} \end{aligned}$$

and

$$\begin{aligned} \pi _{u, CDM, W} = \hat{\pi }_s+z_{\alpha /2}\left[ \frac{p_2^2}{(p_2-p_1)^2}\cdot \frac{\hat{\lambda }_1(1-\hat{\lambda }_1)}{n_1} +\frac{p_1^2}{(p_2-p_1)^2}\cdot \frac{\hat{\lambda }_2(1-\hat{\lambda }_2)}{n_2}\right] ^{1/2}. \end{aligned}$$

Alternately, since $\pi _s$ $=$ $(p_2\lambda _1-p_1\lambda _2)/(p_2-p_1)$, we can first construct the Wilson confidence intervals for $\lambda _1$ and $\lambda _2$ and subsequently obtain the confidence interval for $\pi _s$ via the method of variance estimates recovery (MOVER) proposed by Zou & Donner (2008). It is shown that the $(1-\alpha )100\%$ Wilson confidence lower and upper limits for $\lambda _i$ can be obtained by

23 $$\begin{aligned} l_i=\frac{n_i\hat{\lambda }_i+z_{\alpha /2}^2/2-z_{\alpha /2}\sqrt{n_i\hat{\lambda }_i(1-\hat{\lambda }_i)+z_{\alpha /2}^2/4}}{n_i+z_{\alpha /2}^2}, \text{ and }\end{aligned}$$

24 $$\begin{aligned} u_i=\frac{n_i\hat{\lambda }_i+z_{\alpha /2}^2/2+z_{\alpha /2}\sqrt{n_i\hat{\lambda }_i(1-\hat{\lambda }_i)+z_{\alpha /2}^2/4}}{n_i+z_{\alpha /2}^2} \end{aligned}$$

for *i* = 1,2.

By using MOVER proposed by Zou & Donner (2008), the $(1-\alpha )100\%$ confidence interval for $\pi _s$ is given by

25 $$\begin{aligned} \text{ CI}_{CDM, Wi}=[\pi _{l, CDM, Wi}, \pi _{u, CDM, Wi}], \end{aligned}$$

where

$$\begin{aligned} \pi _{l, CDM, Wi} =\hat{\pi }_s-\frac{1}{|p_2-p_1|}\left[ p_2^2(\hat{\lambda }_1-l_1)^2+p_1^2(u_2-\hat{\lambda }_2)^2\right] ^{1/2} \end{aligned}$$

and

$$\begin{aligned} \pi _{u, CDM, Wi} =\hat{\pi }_s+\frac{1}{|p_2-p_1|}\left[ p_2^2(u_1-\hat{\lambda }_1)^2+p_1^2(\hat{\lambda }_2-l_2)^2\right] ^{1/2}. \end{aligned}$$

#### Sample Size Formula Based on Wald Confidence Interval ${{\varvec{[n_{CDM, W}}}}$, ${{\varvec{n_{CDM, W, 0.5}]}}}$

The half width of the $(1-\alpha )100\%$ Wald CI is given by

$$\begin{aligned} z_{\alpha /2}\left[ \frac{p_2^2}{(p_2-p_1)^2}\cdot \frac{\hat{\lambda }_1(1-\hat{\lambda }_1)}{n_1}+ \frac{p_1^2}{(p_2-p_1)^2}\cdot \frac{\hat{\lambda }_2(1-\hat{\lambda }_2)}{n_2}\right] ^{1/2}. \end{aligned}$$

To control the half width of the Wald CI to be no larger than $\omega$ with probability $1-\beta$, we have

$$\begin{aligned} Pr\left( z_{\alpha /2}\left[ \frac{p_2^2}{(p_2-p_1)^2}\cdot \frac{\hat{\lambda }_1(1-\hat{\lambda }_1)}{n_1}+ \frac{p_1^2}{(p_2-p_1)^2}\cdot \frac{\hat{\lambda }_2(1-\hat{\lambda }_2)}{n_2}\right] ^{1/2}\le \omega \right) \ge 1-\beta . \end{aligned}$$

Let *A* $=$ $p_2^2/n_1$, *B* $=$ $p_1^2/n_2$ and *C* $=$ $\omega |p_2-p_1|/z_{\alpha /2}$, then we have

$$\begin{aligned} Pr(A\hat{\lambda }_1(1-\hat{\lambda }_1)+B\hat{\lambda }_2(1-\hat{\lambda }_2)\le C^2)\ge 1-\beta . \end{aligned}$$

Similarly, it is clear that

$$\begin{aligned} \begin{array}{lll} A\hat{\lambda }_1(1-\hat{\lambda }_1)+B\hat{\lambda }_2(1-\hat{\lambda }_2)\\ \sim N(A\lambda _1(1-\lambda _1)+B\lambda _2(1-\lambda _2), \frac{A^2\lambda _1(1-\lambda _1)(1-2\lambda _1)^2}{n_1}+\frac{B^2\lambda _2(1-\lambda _2)(1-2\lambda _2)^2}{n_2}). \end{array} \end{aligned}$$

Therefore, we have

$$\begin{aligned} \frac{C^2-A\lambda _1(1-\lambda _1)-B\lambda _2(1-\lambda _2)}{\left[ \frac{A^2\lambda _1(1-\lambda _1)(1-2\lambda _1)^2}{n_1}. +\frac{B^2\lambda _2(1-\lambda _2)(1-2\lambda _2)^2}{n_2}\right] ^{1/2}}=z_\beta . \end{aligned}$$

If $n_1$ $=$ $n_2$ $=$ $\frac{1}{2}n$, the above equation can be simplified as

26 $$\begin{aligned} C^4n^3-4C^2Dn^2+4D^2n-8z_\beta ^2E=0, \end{aligned}$$

where $D=p_2^2\lambda _1(1-\lambda _1)+p_1^2\lambda _2(1-\lambda _2)$ and *E* $=$ $p_2^4\lambda _1(1-\lambda _1)(1-2\lambda _1)^2$ $+$ $p_1^4\lambda _2(1-\lambda _2)$ $(1-2\lambda _2)^2$. By using Cardans formula for solving the cubic equation, we can obtain the solutions and the desired sample size is the minimum integer that is not smaller than the unique real root or the maximum root of three real roots. The estimated sample size is denoted as ${{\varvec{n_{CDM, W}}}}$.

When $\beta$ $=$ 0.5, the formula reduces to be

27 $$\begin{aligned} {{\varvec{n_{CDM, W, 0.5}}}}=\frac{2z_{\alpha /2}^2[p_2^2\lambda _1(1-\lambda _1)+p_1^2\lambda _2(1-\lambda _2)]}{\omega ^2(p_2-p_1)^2}. \end{aligned}$$

#### Sample Size Formula Based on Wilson Confidence Interval ${{\varvec{[n_{CDM, Wi}}}}$, ${{\varvec{n_{CDM, Wi, 0.5}]}}}$

The width of the $(1-\alpha )100\%$ Wilson CI is given by

$$\begin{aligned} \frac{1}{|p_2-p_1|}\Big (\sqrt{p_2^2(u_1-\hat{\lambda }_1)^2+p_1^2(\hat{\lambda }_2-l_2)^2}+ \sqrt{p_2^2(\hat{\lambda }_1-l_1)^2+p_1^2(u_2-\hat{\lambda }_2)^2}\Big ). \end{aligned}$$

To control the width of the Wilson CI to be no larger than $2\omega$ with probability $1-\beta$, we have

28 $$\begin{aligned} Pr\left( \frac{1}{|p_2-p_1|}\Big [\sqrt{p_2^2(u_1-\hat{\lambda }_1)^2+p_1^2(\hat{\lambda }_2-l_2)^2}+ \sqrt{p_2^2(\hat{\lambda }_1-l_1)^2+p_1^2(u_2-\hat{\lambda }_2)^2}\Big ]\le 2\omega \right) \ge 1-\beta . \end{aligned}$$

Let *a* $$=$$ $$p_2^2(\bar{u}_1-\lambda _1)^2$$ $$+$$ $$p_1^2(\lambda _2-\bar{l}_2)^2$$, *b* $$=$$ $$p_2^2(\lambda _1-\bar{l}_1)^2$$ $$+$$ $$p_1^2(\bar{u}_2-\lambda _2)^2$$ with

$$\begin{aligned} \bar{l}_i=\frac{n_i\lambda _i+z_{\alpha /2}^2/2-z_{\alpha /2}\sqrt{n_i\lambda _i(1-\lambda _i)+z_{\alpha /2}^2/4}}{n_i+z_{\alpha /2}^2},\\ \bar{u}_i=\frac{n_i\lambda _i+z_{\alpha /2}^2/2+z_{\alpha /2}\sqrt{n_i\lambda _i(1-\lambda _i)+z_{\alpha /2}^2/4}}{n_i+z_{\alpha /2}^2}, \end{aligned}$$

and *c* $=$ $n_1\lambda _1(1-\lambda _1)+z_{\alpha /2}^2/4$, *d* $=$ $n_2\lambda _2(1-\lambda _2)+z_{\alpha /2}^2/4$, *e* $=$ $[p_2^2(u_1-\hat{\lambda }_1)^2+p_1^2(\hat{\lambda }_2-l_2)^2]^{1/2}$ $+$ $[p_2^2(\hat{\lambda }_1-l_1)^2+p_1^2(u_2-\hat{\lambda }_2)^2]^{1/2}$. It is shown that the variance of *e* is given by

$$\begin{aligned} \begin{array}{lll} \sigma _e^2&{}=&{}\left[ \frac{(u_1-\lambda _1)(\frac{n_1(1-2\lambda _1)}{2\sqrt{c}}-z_{\alpha /2})}{\sqrt{a}}+\frac{(\lambda _1-l_1) (\frac{n_1(1-2\lambda _1)}{2\sqrt{c}}+z_{\alpha /2}))}{\sqrt{b}}\right] ^2\cdot \frac{z_{\alpha /2}^2p_2^4}{(n_1+z_{\alpha /2}^2)^2} \cdot \frac{\lambda _1(1-\lambda _1)}{n_1}\\ &{}&{}+\left[ \frac{(\lambda _2-l_2)(\frac{n_2(1-2\lambda _2)}{2\sqrt{d}}+z_{\alpha /2})}{\sqrt{a}}+\frac{(u_2-\lambda _2) (\frac{n_2(1-2\lambda _2)}{2\sqrt{d}}-z_{\alpha /2}))}{\sqrt{b}}\right] ^2\cdot \frac{z_{\alpha /2}^2p_1^4}{(n_2+z_{\alpha /2}^2)^2} \cdot \frac{\lambda _2(1-\lambda _2)}{n_2}, \end{array} \end{aligned}$$

i.e.,

$$\begin{aligned} e\sim N(\sqrt{a}+\sqrt{b},\sigma _e^2). \end{aligned}$$

Equation (28) can be re-written as

$$\begin{aligned} Pr(\frac{e-(\sqrt{a}+\sqrt{b})}{\sigma _e}\le \frac{2\omega |p_2-p_1|-\sqrt{a}+\sqrt{b})}{\sigma _e})\ge 1-\beta . \end{aligned}$$

Therefore, we have

29 $$\begin{aligned} 2\omega |p_2-p_1|-(\sqrt{a}+\sqrt{b})=z_\beta \sigma _e. \end{aligned}$$

When $\beta$ $=$ 0.5, the equation is given as

30 $$\begin{aligned} a+b+2\sqrt{ab}=4\omega ^2(p_2-p_1)^2. \end{aligned}$$

If $n_1$ $=$ $n_2$ $=$ $\frac{1}{2}n$, the desired sample sizes can be obtained by solving Eqs. (29) or (30) via the secant method. The desired sample sizes are estimated to be the minimum integer that is not smaller than the maximum real root of Eqs. (29) or (30), respectively. The corresponding estimated sample sizes (i.e., the values of *n*) are denoted as ${{\varvec{n_{CDM, Wi}}}}$ and ${{\varvec{n_{CDM, Wi, 0.5}}}}$, respectively.

## Evaluation

To evaluate the formulas proposed in this article, we consider the following parameter settings for different models:

(a) Warner model: (1) *p* $=$ 0.3, 0.6, 0.8; (2) $\pi _s$ $=$ 0.04(0.04)0.16; (3) $\omega$ $=$ $25\%$ or $50\%$ of $\pi _s$; i.e., a total of 3 $\times$ 4 $\times$ 2 $=$ 24 parameter combinations.

(b) Unrelated question model: $p=0.75$ and (1) $\pi _N$ $=$ 0.2(0.3)0.8; (2) $\pi _s$ $=$ 0.04(0.04)0.16; (3) $\omega$ $=$ $25\%$ or $50\%$ of $\pi _s$; i.e., a total of 3 $\times$ 4 $\times$ 2 $=$ 24 parameter combinations.

(c) Item count technique: (2) *k* $=$ 4(2)8; (2) $\pi _s$ $=$ 0.04(0.04)0.16; (3) $\pi _i=0.5$ for $i=1,2,\ldots ,k$; (4) $\omega$ $=$ $25\%$ or $50\%$ of $\pi _s$; i.e., a total of 3 $\times$ 4 $\times$ 2 $=$ 24 parameter combinations.

(d) Cheater detection model: (1) $p_1$ $=$ 1/3, $p_2$ $=$ 2/3 or $p_1$ $=$ 1/4, $p_2$ $=$ 3/4; (2) $\beta _s$ $=$ 0.04(0.04)0.16; (3) $\pi _s$ $=$ 0.04(0.04)0.16; (4) $\omega$ $=$ $25\%$ or $50\%$ of $\pi _s$; i.e., a total of 3 $\times$ 4 $\times$ 2 $=$ 64 parameter combinations.

According to the formulas developed in Sect. 1, the desired sample sizes can be estimated for different RRT models. Given the estimated sample sizes, we can then consider their empirical coverage probabilities (ECPs), empirical assurance probabilities (EAPs), left noncoverage probabilities (LNCPs) and right noncoverage probabilities (RNCPs) of the $(1-\alpha )100\%$ Wald and Wilson CIs for evaluating the accuracy of various sample size formulas. For all models, the confidence level $1-\alpha$ is set to be 0.95, and the number of replications is set to be $K=10000$ when calculating the following evaluation indices:

(i) Empirical Assurance Probability (EAP)

$$\begin{aligned} \text{ EAP }=\frac{1}{K}\sum \limits _{k=1}^K I(\pi _{u}^{(k)}-\pi _{l}^{(k)}\le 2\omega ), \end{aligned}$$

where $(\pi _{l}^{(k)},\pi _{u}^{(k)})$ is the CI for $\pi _s$ at the *k*th replication, and $I(\cdot )$ is the indicator function of the event that $\pi _{u}^{(k)}-\pi _{l}^{(k)}\le 2\omega$.

(ii) Empirical Coverage Probability (ECP)

$$\begin{aligned} \text{ ECP }=\frac{1}{K}\sum \limits _{k=1}^K I(\pi _s\in (\pi _{l}^{(k)},\pi _{u}^{(k)})). \end{aligned}$$

(iii) Left and Right Noncoverage Probability (LNCP and RNCP)

$$\begin{aligned} \begin{array}{lll} \text{ LNCP }&{}=&{}\frac{1}{K}\sum \limits _{k=1}^K I(\pi _s\le \pi _{l}^{(k)}),\text{ and }\\ \text{ RNCP }&{}=&{}\frac{1}{K}\sum \limits _{k=1}^K I(\pi _s\ge \pi _{u}^{(k)}). \end{array} \end{aligned}$$

Simulation results for assessing the accuracy of various sample size formulas under Warner’s model, unrelated question model, item count technique and cheater detection model are reported in Tables 1, 2, 3, 4 and 5.

**Table 1:** Performance of the sample size formula with $1-\beta$ assurance probability for Warner’s RRT model under (i) $\beta =0.5$ and (ii) $\beta =0.05$ with *p* = 0.3, 0.6, 0.8

		Wald			Wilson		
$\pi _s$	$\omega ^\flat$	$n^\dag$	ECP(L,R)${\%}^\ddag$	EAP	n	ECP(L,R)$\%$	EAP
(i) $\beta$ $=$ 0.5							
$$p=0.3$$							
0.04	25	51896	94.64(2.75,2.61)	49.66	51893	94.82(2.57,2.61)	48.76
	50	12974	94.96(2.62,2.42)	50.19	12971	94.87(2.73,2.40)	50.36
0.08	25	13312	95.03(2.43,2.54)	50.27	13309	94.48(2.82,2.70)	50.45
	50	3328	94.63(2.49,2.88)	50.16	3325	94.92(2.37,2.71)	50.93
0.12	25	6053	94.69(2.49,2.82)	50.98	6050	95.01(2.42,2.57)	51.35
	50	1513	95.48(2.31,2.21)	50.46	1510	95.14(2.81,2.05)	50.58
0.16	25	3474	94.82(2.55,2.63)	50.61	3471	94.70(2.77,2.53)	50.19
	50	868	94.93(2.39,2.68)	48.72	865	95.08(2.45,2.47)	48.24
$$p=0.6$$							
0.04	25	231971	94.83(2.41,2.76)	49.55	231968	94.83(2.52,2.65)	49.49
	50	57992	95.35(2.23,2.42)	50.30	57990	95.03(2.54,2.43)	50.91
0.08	25	58330	95.07(2.62,2.31)	49.55	58328	95.05(2.42,2.53)	49.66
	50	14582	95.12(2.60,2.28)	50.08	14579	94.88(2.74,2.38)	49.52
0.12	25	26061	95.10(2.64,2.26)	50.21	26058	95.20(2.52,2.28)	50.59
	50	6515	94.88(2.65,2.47)	49.68	6512	95.05(2.51,2.44)	50.52
0.16	25	14728	94.83(2.40,2.77)	49.28	14725	95.21(2.41,2.38)	49.91
	50	3682	95.06(2.22,2.72)	49.69	3679	95.13(2.35,2.52)	51.51
$$p=0.8$$							
0.04	25	18548	94.90(2.60,2.50)	49.74	18547	95.04(2.65,2.31)	49.97
	50	4637	95.29(2.16,2.55)	50.37	4636	95.22(2.41,2.37)	51.12
0.08	25	4975	95.06(2.30,2.64)	49.95	4973	94.93(2.58,2.49)	50.77
	50	1243	95.24(2.14,2.62)	47.83	1242	94.79(2.82,2.39)	51.88
0.12	25	2347	94.88(2.31,2.81)	50.49	2346	95.25(2.44,2.31)	51.59
	50	586	95.02(1.89,3.09)	47.15	585	95.12(2.24,2.64)	51.70
0.16	25	1389	94.79(2.35,2.86)	49.35	1387	94.73(2.88,2.39)	49.95
	50	347	95.31(1.91,2.78)	49.66	345	95.08(2.60,2.32)	53.50
(ii) $\beta$ $=$ 0.05							
$$p=0.3$$							
0.04	25	52192	95.00(2.53,2.47)	95.03	52188	94.78(2.66,2.56)	94.39
	50	13121	94.78(2.64,2.58)	95.10	13118	94.85(2.62,2.53)	95.19
0.08	25	13447	94.80(2.55,2.65)	95.16	13443	94.93(2.66,2.41)	94.98
	50	3395	95.20(2.04,2.76)	95.90	3391	95.00(2.44,2.56)	95.63
0.12	25	6134	95.04(2.34,2.62)	95.61	6130	95.07(2.35,2.58)	95.51
	50	1553	94.85(2.35,2.80)	96.20	1550	95.61(2.17,2.22)	96.33
0.16	25	3528	94.73(2.65,2.62)	95.45	3524	94.89(2.70,2.41)	95.32
	50	895	95.26(2.22,2.52)	96.46	891	95.18(2.37,2.45)	96.23
$$p=0.6$$							
0.04	25	232267	95.07(2.60,2.33)	95.01	232263	94.71(2.55,2.74)	94.99
	50	58141	95.02(2.34,2.64)	95.40	58137	94.94(2.47,2.59)	95.45
0.08	25	58466	95.03(2.56,2.41)	95.09	58462	94.80(2.69,2.51)	95.02
	50	14650	95.31(2.55,2.14)	95.42	14646	95.10(2.69,2.21)	95.38
0.12	25	26143	95.46(2.28,2.26)	95.69	26139	95.06(2.50,2.44)	95.35
	50	6556	94.81(2.60,2.59)	95.99	6552	95.01(2.57,2.42)	95.80
0.16	25	14783	95.19(2.33,2.48)	95.51	14779	95.11(2.22,2.67)	95.91
	50	3709	95.00(2.38,2.62)	96.40	3705	95.18(2.44,2.38)	96.29
$$p=0.8$$							
0.04	25	18844	95.16(2.40,2.44)	94.87	18841	94.46(2.85,2.69)	94.93
	50	4784	94.93(2.11,2.96)	95.72	4780	95.46(2.22,2.32)	95.43
0.08	25	5109	95.17(2.21,2.62)	95.48	5106	95.54(2.19,2.27)	95.47
	50	1310	95.17(2.11,2.72)	95.65	1307	95.09(2.67,2.24)	95.38
0.12	25	2428	95.18(1.91,2.91)	95.77	2425	95.28(2.35,2.37)	95.55
	50	627	95.02(1.96,3.02)	96.36	623	95.20(2.57,2.23)	95.77
0.16	25	1444	94.89(2.15,2.96)	95.83	1440	94.94(2.55,2.51)	95.94
	50	374	94.77(2.30,2.93)	97.04	370	94.69(3.04,2.27)	96.24

$^\flat$ Half width (i.e., $\omega$) of a CI as given by the value of $\pi _s$, i.e., $25\%$ and $50\%$ of $\pi _s$.

$^\dag$ *n* denotes the estimated sample size;    $^\ddag$ (L,R) denotes (LNCP, RNCP).

**Table 2:** Performance of the sample size formula with $1-\beta$ assurance probability for UQM under (i) $\beta =0.5$ and (ii) $\beta = 0.05$ with $\pi _N$ = 0.2, 0.5, 0.8

		Wald			Wilson		
$\pi _s$	$\omega$	*n*	ECP(L,R)$\%$	EAP	*n*	ECP(L,R)$\%$	EAP
(i) $\beta$ $=$ 0.5							
$\pi _N$ $=$ 0.2							
0.04	25	5026	94.84(2.12,3.04)	51.47	5032	94.75(2.71,2.54)	50.10
	50	1256	94.12(2.19,3.69)	50.09	1262	94.38(3.11,2.51)	48.65
0.08	25	1671	94.80(1.85,3.35)	49.57	1674	95.12(2.35,2.53)	51.59
	50	417	94.99(1.91,3.10)	48.74	420	95.06(3.00,1.94)	53.04
0.12	25	913	94.70(2.07,3.23)	49.13	914	94.96(2.64,2.40)	48.46
	50	228	93.43(1.62,4.95)	47.88	229	95.57(2.53,1.90)	53.86
0.16	25	602	94.75(2.30,2.95)	51.60	602	95.30(2.54,2.16)	51.32
	50	150	94.85(1.80,3.35)	50.23	150	95.13(3.00,1.87)	50.54
$$\pi _N=0.5$$							
0.04	25	8944	94.97(2.54,2.49)	50.21	8945	94.92(2.64,2.44)	49.79
	50	2236	94.56(2.32,3.12)	49.96	2236	95.01(2.51,2.48)	50.35
0.08	25	2574	95.17(2.15,2.68)	51.16	2573	94.73(2.67,2.60)	51.46
	50	643	95.17(1.90,2.93)	48.96	643	95.28(2.47,2.25)	52.76
0.12	25	1280	95.12(1.99,2.89)	48.48	1279	94.91(2.75,2.34)	52.18
	50	320	94.78(1.82,3.40)	48.95	319	94.38(3.03,2.59)	50.15
0.16	25	789	94.97(2.21,2.82)	50.61	788	95.06(2.53,2.41)	51.37
	50	197	94.09(2.52,3.39)	51.85	195	94.16(2.81,3.03)	48.07
$$\pi _N=0.8$$							
0.04	25	12095	94.92(2.49,2.59)	50.50	12093	95.16(2.59,2.25)	51.06
	50	3023	94.77(2.38,2.85)	50.65	3022	95.13(2.36,2.51)	51.24
0.08	25	3284	94.97(2.23,2.80)	49.06	3283	95.02(2.52,2.46)	49.48
	50	821	94.85(2.40,2.75)	50.76	819	94.65(2.59,2.76)	52.99
0.12	25	1562	94.62(2.62,2.76)	49.64	1560	94.83(2.66,2.51)	50.85
	50	390	94.53(2.22,3.25)	47.84	388	95.04(2.39,2.57)	50.30
0.16	25	928	95.17(2.41,2.42)	49.34	926	95.44(2.44,2.12)	51.10
	50	232	94.55(2.14,3.31)	52.07	229	95.32(2.32,2.36)	52.01
(ii) $\beta$ $=$ 0.05							
$$\pi _N=0.2$$							
0.04	25	5381	94.47(2.38,3.15)	94.70	5380	94.77(2.63,2.60)	95.02
	50	1431	95.30(1.77,2.93)	95.58	1429	95.05(2.57,2.38)	95.17
0.08	25	1835	94.46(2.20,3.34)	95.12	1832	94.40(2.92,2.68)	94.78
	50	498	94.42(2.12,3.46)	96.17	495	95.65(2.53,1.82)	95.54
0.12	25	1014	94.83(2.15,3.02)	95.79	1011	95.48(2.29,2.23)	95.54
	50	277	95.19(1.43,3.38)	96.88	274	94.89(2.78,2.33)	95.87
0.16	25	671	94.77(2.00,3.23)	96.03	668	95.27(2.66,2.07)	95.74
	50	184	94.70(1.62,3.68)	96.29	180	95.58(2.59,1.83)	95.79
$$\pi _N=0.5$$							
0.04	25	9239	95.19(2.19,2.62)	95.38	9236	95.10(2.52,2.38)	95.02
	50	2382	95.43(2.00,2.57)	95.45	2379	95.14(2.40,2.46)	95.47
0.08	25	2708	94.85(2.42,2.73)	95.43	2704	94.54(2.92,2.54)	94.48
	50	709	94.75(1.89,3.36)	96.09	706	95.29(2.70,2.01)	95.85
0.12	25	1361	94.76(2.02,3.22)	95.50	1357	95.00(2.38,2.62)	95.59
	50	359	94.67(1.98,3.35)	96.70	356	94.52(2.92,2.56)	96.23
0.16	25	843	94.68(2.26,3.06)	96.34	839	94.47(3.03,2.50)	95.20
	50	223	94.68(1.63,3.69)	96.81	220	95.11(2.67,2.22)	96.51
$$\pi _N=0.8$$							
0.04	25	12326	95.22(2.38,2.40)	95.21	12322	94.85(2.59,2.56)	95.18
	50	3138	94.76(2.39,2.85)	95.48	3135	94.99(2.45,2.56)	95.09
0.08	25	3387	95.22(2.12,2.66)	95.80	3383	95.05(2.46,2.49)	95.64
	50	872	94.32(2.46,3.22)	95.93	868	94.81(2.52,2.67)	95.38
0.12	25	1622	94.94(2.47,2.59)	96.29	1618	95.01(2.77,2.22)	95.58
	50	420	94.73(2.25,3.02)	96.26	416	94.65(3.01,2.34)	96.24
0.16	25	967	94.84(2.64,2.52)	95.94	963	95.33(2.60,2.07)	95.85
	50	251	95.04(1.97,2.99)	97.20	247	95.41(2.49,2.10)	97.51

**Table 3:** Performance of the sample size formula with $1-\beta$ assurance probability for ICT model under (i) $\beta =0.5$ and (ii) $\beta =0.05$ with *k* = 4, 6, 8

		Wald			Wilson		
$\pi _s$	$\omega$	*n*	ECP(L,R)$\%$	EAP	*n*	ECP(L,R)$\%$	EAP
(i) $\beta$ $=$ 0.5							
*k* $=$ 4							
0.04	25	156614	95.25(2.41,2.34)	49.16	156608	95.04(2.33,2.63)	50.43
	50	39152	94.99(2.43,2.58)	49.88	39148	94.99(2.51,2.50)	50.53
0.08	25	39828	94.65(2.79,2.56)	49.23	39824	94.83(2.83,2.34)	50.04
	50	9956	94.66(2.53,2.81)	49.98	9952	94.77(2.47,2.76)	51.49
0.12	25	17974	95.03(2.32,2.65)	50.84	17970	94.98(2.23,2.79)	50.47
	50	4492	94.71(2.56,2.73)	49.25	4488	94.76(2.58,2.66)	50.75
0.16	25	10248	94.83(2.53,2.64)	48.80	10244	95.26(2.47,2.27)	51.64
	50	2562	95.34(2.11,2.55)	48.85	2556	95.40(2.04,2.56)	50.67
$$k=6$$							
0.04	25	233446	94.78(2.78,2.44)	49.30	233440	95.06(2.53,2.41)	50.47
	50	58360	94.70(2.56,2.74)	49.58	58356	94.94(2.42,2.64)	50.54
0.08	25	59036	94.77(2.49,2.74)	49.48	59032	94.89(2.52,2.59)	50.40
	50	14758	94.99(2.35,2.66)	48.72	14754	94.94(2.63,2.43)	50.82
0.12	25	26512	95.03(2.56,2.41)	50.48	26506	94.84(2.48,2.68)	50.54
	50	6628	94.81(2.49,2.70)	50.33	6622	94.89(2.45,2.66)	50.83
0.16	25	15050	94.77(2.54,2.69)	49.94	15044	95.12(2.44,2.44)	49.81
	50	3762	94.96(2.33,2.71)	49.48	3756	95.08(2.33,2.59)	50.71
$$k=8$$							
0.04	25	310278	95.36(1.99,2.65)	50.76	310272	94.97(2.49,2.54)	50.35
	50	77568	95.33(2.42,2.25)	49.78	77564	95.11(2.40,2.49)	49.76
0.08	25	78244	95.02(2.66,2.32)	49.49	78240	95.35(2.30,2.35)	50.72
	50	19560	95.09(2.52,2.39)	49.44	19556	94.90(2.48,2.62)	51.27
0.12	25	35048	95.11(2.24,2.65)	49.71	35042	95.08(2.30,2.62)	50.08
	50	8762	95.32(2.15,2.53)	50.24	8756	94.84(2.59,2.57)	50.87
0.16	25	19852	95.02(2.69,2.29)	48.45	19846	94.93(2.58,2.49)	49.08
	50	4962	95.11(2.41,2.48)	47.55	4956	95.11(2.54,2.35)	50.80
(ii) $\beta$ $=$ 0.05							
$$k=4$$							
0.04	25	157206	94.83(2.66,2.51)	95.16	157198	95.20(2.38,2.42)	95.29
	50	39448	94.99(2.46,2.55)	95.15	39442	94.98(2.70,2.32)	95.11
0.08	25	40098	94.78(2.52,2.70)	95.06	40092	94.46(2.88,2.66)	94.97
	50	10090	94.93(2.38,2.69)	95.61	10084	94.68(2.38,2.94)	95.58
0.12	25	18136	95.06(2.36,2.58)	95.58	18130	94.55(2.59,2.86)	95.43
	50	4574	95.30(2.41,2.29)	95.93	4566	95.14(2.42,2.44)	96.08
0.16	25	10358	94.85(2.49,2.66)	95.87	10350	95.06(2.56,2.38)	95.80
	50	2616	95.15(2.13,2.72)	96.61	2608	95.28(2.23,2.49)	96.45
$$k=6$$							
0.04	25	234038	94.88(2.66,2.46)	94.87	234030	95.25(2.45,2.30)	95.06
	50	58656	95.19(2.42,2.39)	95.35	58650	94.99(2.50,2.51)	95.22
0.08	25	59306	95.04(2.49,2.47)	95.02	59300	94.80(2.59,2.61)	95.11
	50	14894	94.76(2.51,2.73)	95.58	14886	94.87(2.61,2.52)	95.75
0.12	25	26674	94.98(2.55,2.47)	95.13	26666	95.02(2.60,2.38)	95.41
	50	6708	94.84(2.54,2.62)	96.05	6700	95.36(2.25,2.39)	96.11
0.16	25	15160	94.72(2.72,2.56)	95.37	15152	94.78(2.69,2.53)	95.35
	50	3816	94.59(2.50,2.91)	96.28	3808	94.82(2.60,2.58)	95.97
$$k=8$$							
0.04	25	310870	94.99(2.47,2.54)	94.99	310862	95.11(2.46,2.43)	95.13
	50	77864	95.08(2.53,2.39)	95.04	77858	94.96(2.54,2.50)	95.21
0.08	25	78516	95.07(2.45,2.48)	95.43	78508	95.15(2.46,2.39)	95.39
	50	19696	94.88(2.72,2.40)	95.29	19688	94.96(2.46,2.58)	95.42
0.12	25	35212	94.98(2.47,2.55)	95.55	35204	94.84(2.42,2.74)	95.51
	50	8842	94.85(2.37,2.78)	95.69	8836	94.60(2.80,2.60)	95.65
0.16	25	19962	94.48(2.74,2.78)	95.68	19954	95.05(2.46,2.49)	95.40
	50	5016	95.06(2.49,2.45)	96.19	5010	95.00(2.63,2.37)	96.52

**Table 4:** Performance of the sample size formula with $1-\beta$ assurance probability for CDM for ($p_1$, $p_2$) $=$ (1/3, 2/3) under (i) $\beta =0.5$ and (ii) $\beta =0.05$ with $\beta _s$ = 0.04, 0.08, 0.12, 0.16

		Wald			Wilson		
$\pi _s$	$\omega$	*n*	ECP(L,R)$\%$	EAP	*n*	ECP(L,R)$\%$	EAP
(i) $\beta$ $=$ 0.5							
$\beta _s$ $=$ 0.04							
0.04	25	20296	95.20(2.36,2.44)	49.89	20336	94.83(2.43,2.74)	51.00
	50	5074	94.90(1.90,3.20)	50.31	5114	95.29(2.45,2.26)	50.82
0.08	25	8332	94.86(2.22,2.92)	50.32	8348	95.13(2.43,2.44)	50.09
	50	2082	94.78(2.21,3.01)	50.87	2098	94.51(3.04,2.45)	50.11
0.12	25	5014	95.26(2.16,2.58)	50.80	5020	95.00(2.67,2.33)	49.91
	50	1252	94.91(2.15,2.94)	50.18	1258	95.33(2.66,2.01)	50.41
0.16	25	3480	95.09(2.28,2.63)	50.08	3482	95.07(2.52,2.41)	50.05
	50	870	95.18(1.93,2.89)	51.17	870	95.24(2.50,2.26)	49.18
$\beta _s$ $=$ 0.08							
0.04	25	25624	95.04(2.11,2.85)	50.28	25652	94.79(2.45,2.76)	50.44
	50	6406	94.96(2.38,2.66)	49.40	6432	94.90(2.76,2.34)	49.79
0.08	25	9540	94.90(2.42,2.68)	50.05	9552	94.96(2.65,2.39)	50.92
	50	2384	94.91(2.09,3.00)	49.93	2396	94.95(2.82,2.23)	49.99
0.12	25	5496	95.17(2.11,2.72)	50.48	5500	94.93(2.75,2.32)	50.02
	50	1374	95.27(2.07,2.66)	49.98	1378	95.12(2.55,2.33)	50.84
0.16	25	3722	94.82(2.39,2.79)	50.79	3722	95.43(2.38,2.19)	50.93
	50	930	94.67(2.21,3.12)	49.78	930	94.52(2.80,2.68)	50.72
$\beta _s$ $=$ 0.12							
0.04	25	30732	94.68(2.38,2.94)	51.28	30752	94.74(2.72,2.54)	50.16
	50	7682	95.02(2.39,2.59)	49.50	7702	94.92(2.75,2.33)	49.89
0.08	25	10694	94.63(2.50,2.87)	49.34	10702	94.89(2.56,2.55)	49.83
	50	2672	95.13(2.17,2.70)	50.11	2680	95.16(2.38,2.46)	50.74
0.12	25	5954	94.81(2.50,2.69)	49.59	5956	95.00(2.47,2.53)	49.67
	50	1488	95.26(2.02,2.72)	50.25	1490	95.30(2.37,2.33)	50.80
0.16	25	3948	94.99(2.25,2.76)	49.82	3946	95.16(2.44,2.40)	49.51
	50	986	94.71(2.21,3.08)	50.16	986	95.59(2.42,1.99)	51.10
$\beta _s$ $=$ 0.16							
0.04	25	35622	94.87(2.44,2.69)	50.22	35636	94.60(2.57,2.83)	50.43
	50	8904	94.70(2.65,2.65)	50.59	8918	94.76(2.84,2.40)	49.50
0.08	25	11794	94.99(2.39,2.62)	49.94	11798	95.25(2.36,2.39)	49.18
	50	2948	95.31(2.09,2.60)	49.72	2952	94.93(2.51,2.56)	49.97
0.12	25	6388	95.28(2.19,2.53)	50.02	6390	94.97(2.71,2.32)	50.05
	50	1596	95.18(2.01,2.81)	50.03	1596	94.71(2.66,2.63)	49.89

		Wald			Wilson		
$\pi _s$	$\omega$	n	ECP(L,R)$\%$	EAP	n	ECP(L,R)$\%$	EAP
0.16	25	4162	94.98(2.23,2.79)	50.03	4160	94.86(2.79,2.35)	50.27
	50	1040	94.73(2.26,3.01)	50.24	1038	94.70(2.41,2.89)	50.57
$\beta$ $=$ 0.05							
(ii) $\beta _s$ $=$ 0.04							
0.04	25	21314	94.87(2.43,2.70)	95.11	21362	94.69(2.63,2.68)	95.29
	50	5572	95.43(1.93,2.64)	94.91	5616	94.78(2.83,2.39)	95.44
0.08	25	8796	95.29(2.21,2.50)	95.18	8816	94.92(2.62,2.46)	95.33
	50	2310	94.61(1.79,3.60)	95.24	2326	95.14(2.38,2.48)	95.30
0.12	25	5294	94.87(2.48,2.65)	95.35	5302	95.01(2.55,2.44)	95.40
	50	1390	95.17(2.02,2.81)	95.29	1396	95.18(2.63,2.19)	95.24
0.16	25	3668	94.88(2.31,2.81)	95.06	3670	94.63(2.72,2.65)	95.29
	50	962	94.42(2.27,3.31)	95.37	962	94.98(2.55,2.47)	95.68
$\beta _s$ $=$ 0.08							
0.04	25	26606	95.13(2.32,2.55)	94.92	26640	95.25(2.44,2.31)	95.24
	50	6888	95.31(2.27,2.42)	94.85	6920	95.18(2.61,2.21)	94.89
0.08	25	9988	95.09(2.38,2.53)	95.16	10004	94.84(2.76,2.40)	94.85
	50	2604	94.63(2.26,3.11)	95.11	2616	94.84(2.43,2.73)	95.58
0.12	25	5766	95.26(2.20,2.54)	95.34	5772	94.75(2.41,2.84)	95.28
	50	1506	95.06(2.01,2.93)	95.30	1510	94.83(2.62,2.55)	95.58
0.16	25	3900	94.83(2.32,2.85)	95.32	3902	95.02(2.44,2.54)	95.70
	50	1018	94.98(1.96,3.06)	95.35	1018	94.99(2.56,2.45)	96.15
$\beta _s$ $=$ 0.12							
0.04	25	31680	94.74(2.66,2.60)	94.92	31706	94.86(2.49,2.65)	95.64
	50	8150	94.89(2.13,2.98)	94.91	8174	95.12(2.56,2.32)	95.34
0.08	25	11126	95.21(2.28,2.51)	95.31	11136	94.82(2.65,2.53)	95.19
	50	2884	94.75(2.24,3.01)	95.21	2894	94.89(2.67,2.44)	95.68
0.12	25	6212	94.90(2.35,2.75)	94.93	6216	94.48(3.00,2.52)	94.93
	50	1614	94.66(2.22,3.12)	95.26	1618	95.25(2.39,2.36)	95.90
0.16	25	4120	94.54(2.63,2.83)	94.96	4120	95.04(2.63,2.33)	95.01
	50	1070	95.03(2.25,2.72)	95.57	1070	94.87(2.51,2.62)	96.00
$\beta _s$ $=$ 0.16							
0.04	25	36536	95.28(2.21,2.51)	95.03	36556	95.35(2.32,2.33)	95.54
	50	9356	94.78(2.44,2.78)	94.93	9374	95.02(2.53,2.45)	95.23
0.08	25	12208	95.17(2.23,2.60)	95.09	12216	95.26(2.50,2.24)	95.05
	50	3152	94.78(2.44,2.78)	95.12	3158	95.09(2.63,2.28)	95.28
0.12	25	6636	94.78(2.75,2.47)	95.12	6638	94.66(2.90,2.44)	95.48
	50	1718	94.81(2.29,2.90)	95.24	1720	95.03(2.69,2.28)	95.74
0.16	25	4324	95.18(2.16,2.66)	94.97	4324	95.18(2.40,2.42)	95.44
	50	1120	95.00(2.18,2.82)	95.44	1118	94.54(2.77,2.69)	95.59

**Table 5:** Performance of the sample size formula with $1-\beta$ assurance probability for CDM for ($p_1$, $p_2$) $=$ (1/4, 3/4) under (i) $\beta =0.5$ and (ii) $\beta =0.05$ with $\beta _s$=0.04, 0.08, 0.12, 0.16

		Wald			Wilson		
$\pi _s$	$\omega$	n	ECP(L,R)$\%$	EAP	n	ECP(L,R)$\%$	EAP
(i) $\beta$ $=$ 0.5							
$\beta _s$ $=$ 0.04							
0.04	25	9460	94.79(1.96,3.25)	51.11	9496	94.91(2.74,2.35)	50.57
	50	2364	94.69(1.80,3.51)	51.19	2400	95.12(2.72,2.16)	51.40
0.08	25	4008	95.39(2.05,2.56)	50.17	4022	94.99(2.92,2.09)	49.58
	50	1002	94.92(1.62,3.46)	50.56	1014	94.74(3.12,2.14)	49.33
0.12	25	2444	95.33(1.97,2.70)	50.20	2448	95.21(2.76,2.03)	50.29
	50	610	94.38(1.89,3.73)	49.94	614	94.97(2.92,2.11)	49.51
0.16	25	1708	94.87(2.24,2.89)	49.69	1708	95.07(2.61,2.32)	50.47
	50	426	94.43(1.92,3.65)	49.96	426	95.66(2.53,1.81)	50.09
$\beta _s$ $=$ 0.08							
0.04	25	11478	95.27(2.02,2.71)	50.05	11504	95.19(2.74,2.07)	49.55
	50	2868	94.88(1.70,3.42)	51.20	2896	95.06(2.63,2.31)	51.02
0.08	25	4466	94.80(2.17,3.03)	50.07	4476	95.06(2.50,2.44)	50.01
	50	1116	94.36(1.99,3.65)	50.83	1126	94.98(2.70,2.32)	51.08
0.12	25	2626	95.19(2.01,2.80)	50.07	2630	95.21(2.56,2.23)	49.88
	50	656	94.93(1.63,3.44)	50.55	658	95.50(2.51,1.99)	50.15
0.16	25	1800	94.74(2.29,2.97)	49.89	1798	94.83(2.55,2.62)	49.78
	50	450	94.47(1.99,3.54)	50.09	448	94.98(2.59,2.43)	49.01
$\beta _s$ $=$ 0.12							
0.04	25	13426	95.32(2.05,2.63)	50.51	13446	95.01(2.46,2.53)	50.00
	50	3356	94.95(2.02,3.03)	50.29	3376	95.44(2.42,2.14)	50.01
0.08	25	4908	95.10(2.06,2.84)	50.23	4916	94.90(2.64,2.46)	50.23
	50	1226	94.60(2.10,3.30)	49.68	1234	95.04(2.73,2.23)	50.54
0.12	25	2802	95.36(2.07,2.57)	50.00	2804	95.04(2.51,2.45)	49.85
	50	700	94.89(1.70,3.41)	50.98	702	94.81(2.86,2.33)	50.84
0.16	25	1886	95.19(2.27,2.54)	50.00	1886	95.02(2.73,2.25)	50.18
	50	470	94.83(1.93,3.24)	48.93	470	95.30(2.55,2.15)	51.08
$\beta _s$ $=$ 0.16							
0.04	25	15304	94.98(2.30,2.72)	51.29	15320	95.20(2.46,2.34)	50.54
	50	3826	95.11(2.04,2.85)	51.35	3840	94.89(2.56,2.55)	50.69
0.08	25	5332	94.98(2.16,2.86)	50.49	5336	95.09(2.51,2.40)	50.46
	50	1332	94.92(1.94,3.14)	50.16	1338	94.84(2.82,2.34)	50.44
0.12	25	2970	94.93(2.19,2.88)	50.39	2970	94.89(2.52,2.59)	50.09
	50	742	94.55(2.22,3.23)	49.76	742	94.98(2.83,2.19)	50.45

		Wald			Wilson		
$\pi _s$	$\omega$	n	ECP(L,R)$\%$	EAP	n	ECP(L,R)$\%$	EAP
0.16	25	1970	95.02(2.19,2.79)	50.76	1968	94.84(2.62,2.54)	50.57
	50	492	94.80(1.66,3.54)	50.41	490	94.97(2.80,2.23)	50.33
(ii) $\beta$ $=$ 0.05							
$\beta _s$ $=$ 0.04							
0.04	25	10246	94.96(2.24,2.80)	95.05	10292	94.87(2.58,2.55)	95.39
	50	2744	94.73(1.86,3.41)	94.59	2784	94.72(3.11,2.17)	95.03
0.08	25	4368	94.93(2.09,2.98)	94.86	4386	95.03(2.81,2.16)	95.33
	50	1174	95.09(1.74,3.17)	95.17	1188	95.36(2.57,2.07)	95.51
0.12	25	2660	95.10(2.18,2.72)	94.89	2668	95.01(2.58,2.41)	95.67
	50	714	94.44(2.09,3.47)	95.42	720	94.85(2.66,2.49)	95.88
0.16	25	1854	94.86(2.13,3.01)	95.55	1856	95.01(2.45,2.54)	95.78
	50	496	94.89(1.85,3.26)	95.31	496	94.67(2.85,2.48)	95.59
$\beta _s$ $=$ 0.08							
0.04	25	12244	95.45(1.84,2.71)	95.38	12278	95.05(2.80,2.15)	95.15
	50	3240	94.03(2.25,3.72)	94.75	3272	94.74(2.72,2.54)	95.62
0.08	25	4816	94.78(2.10,3.12)	95.23	4830	94.97(2.70,2.33)	95.28
	50	1286	95.21(1.77,3.02)	95.33	1296	95.18(2.57,2.25)	95.57
0.12	25	2838	94.96(2.29,2.75)	95.09	2842	94.78(2.64,2.58)	95.08
	50	758	94.98(1.76,3.26)	95.31	762	95.07(2.63,2.30)	95.81
0.16	25	1940	94.64(2.41,2.95)	95.27	1942	94.96(2.51,2.53)	95.81
	50	518	94.82(1.91,3.27)	95.79	516	95.06(2.70,2.24)	95.82
$\beta _s$ $=$ 0.12							
0.04	25	14172	94.94(2.25,2.81)	94.99	14200	94.84(2.48,2.68)	95.77
	50	3720	95.29(1.82,2.89)	94.91	3744	94.90(2.72,2.38)	95.43
0.08	25	5248	94.68(2.39,2.93)	94.84	5260	94.89(2.54,2.57)	95.58
	50	1392	94.93(1.76,3.31)	95.16	1400	95.41(2.47,2.12)	95.65
0.12	25	3006	95.05(2.17,2.78)	95.06	3010	94.90(2.61,2.49)	95.44
	50	800	94.67(1.77,3.56)	95.31	802	94.80(2.67,2.53)	95.93
0.16	25	2024	95.00(2.05,2.95)	95.68	2024	94.83(2.69,2.48)	95.55
	50	538	94.70(1.95,3.35)	96.08	536	94.91(2.94,2.15)	95.89
$\beta _s$ $=$ 0.16							
0.04	25	16032	95.13(2.07,2.80)	95.30	16054	94.99(2.44,2.57)	95.31
	50	4182	94.37(2.30,3.33)	94.63	4202	95.00(2.65,2.35)	95.23
0.08	25	5662	94.94(2.18,2.88)	94.85	5672	94.94(2.67,2.39)	95.35
	50	1494	94.75(1.95,3.30)	95.46	1500	95.25(2.50,2.25)	95.79
0.12	25	3168	94.74(2.13,3.13)	94.97	3172	94.68(2.84,2.48)	95.35
	50	838	94.78(1.98,3.24)	95.42	840	94.71(2.75,2.54)	96.03
0.16	25	2102	94.77(2.35,2.88)	95.42	2100	94.96(2.52,2.52)	95.80
	50	556	94.51(2.14,3.35)	96.08	554	94.85(2.80,2.35)	96.11

When the assurance probability is 95%, results in Tables 1, 2, 3, 4 and 5 consistently show that all the empirical assurance probabilities (EAPs) are generally close to the pre-determined nominal level for both Wald and Wilson methods under the four RRT models. Only when assurance probability is 50%, EAPs of Wald method are slightly lower than the nominal in some cases (e.g., $\pi _s$ $=$ 0.08, 0.12 with *p* $=$ 0.8 and $\pi _s$ $=$ 0.12 with $\pi _N$ $=$ 0.2, 0.8 for Warner’s RRT model). Based on the estimated sample sizes, all CIs perform well in the regard that their ECPs are pretty close to the pre-specified nominal level (i.e., $95\%$) and have satisfactory balance between left- and right-tailed errors. The results also show that the sample size estimates, using Wilson approach, are slightly smaller than those based on Wald approach. And, the estimates are more accurate in terms of actual assurance probabilities, coverage probabilities and balances between left- and right-tailed errors.

The above simulation studies are based on the assumption that the expected prevalence equals the true prevalence. We also conduct simulation studies to investigate the performance of the proposed methods in situations where this assumption does not hold true. For this purpose, we let $\alpha =0.05$, $\beta =0.05$, and assume the true prevalence $\pi _s=0.165$, the expected prevalence $\pi _{es}=r\pi _s$ with $r=0.5,0.7,0.95,1.2$, and the half-widths of CI $\omega =0.05,0.10$. In addition, for cheater detection model, we set the true prevalence $\pi _s=0.165$ and the expected prevalence $\pi _{es}=r_1\pi _s$ with $r_1=0.6,0.95,1.2$. We also consider that the expected cheating parameter $\gamma$ is different from the true parameter. Since $\pi _s+\beta _s+\gamma =1.0$ (i.e., $\gamma =1.0-\pi _s-\beta _s$), we consider the following expected proportion of honest-no respondent (i.e.,$\beta _{es}$), which differs from the true proportion (i.e., $\beta _s$): the true proportion $\beta _s=0.7$ and the expected proportion $\beta _{es}=r_2\beta _s$ with $r_2=0.6,0.95,1.1$. As shown in Tables 1, 2, 3, 4 and 5, the performances of the estimated sample sizes under various models are not affected by the settings of the other parameters (i.e., *p*, $\pi _N$, *k*, $p_1$, $p_2$). Therefore, we consider other settings of the parameters for each model: (i) Warner model: $p=0.3$; (ii) unrelated question model: $p=0.7$ and $\pi _N=0.5$; (iii) item count technique: *k* $=$ 4 and $\pi _i=0.5$ for $i=1,2,\cdots ,k$; (iv) cheater detection model: $p_1$ $=$ 0.2, $p_2$ $=$ 0.8. We conduct the simulation study as follows. First, given the expected prevalence $\pi _{es}$, the expected proportion $\beta _{es}$ and other parameters from each model, the estimated sample size for each model is obtained via the formulas illustrated in Sect. 1. Second, based on the estimated sample size for each model, 10000 random samples are generated under the true parameter values, and consequently 10000 confidence intervals for $\pi _s$ are obtained. Third, based on the 10000 confidence intervals for each model, we calculate the proportion of these intervals that include the true prevalence $\pi _s$ to obtain the ECP, and we calculate the proportion of the widths of the intervals being controlled within the pre-given value (i.e., $\omega$) to obtain the ACP. Simulation results are reported in Table 6.

**Table 6:** Performance of the sample size formula with $95\%$ assurance probability for various models when the expected prevalence or proportion (i.e., $\pi _{es}$ or $\beta _{es}$) differs from the true prevalence or proportion (i.e., $\pi _{s}$ or $\beta _s$)

			Wald			Wilson		
$\omega$	$\pi _{es}^c$	$\beta _{es}^d$	n	ECP(L,R)$\%$	EAP	n	ECP(L,R)$\%$	EAP
		Warner’s RRT						
0.05	0.50	–	2187	95.23(2.41,2.36)	7.00	2183	95.35(2.42,2.23)	6.75
	0.70	–	2224	94.87(2.47,2.66)	44.00	2220	95.08(2.41,2.51)	43.60
	0.95	–	2265	94.83(2.52,2.65)	92.71	2261	95.00(2.62,2.38)	92.99
	1.20	–	2300	94.86(2.53,2.61)	99.94	2296	94.92(2.44,2.64)	99.81
0.10	0.50	–	560	94.69(2.51,2.80)	59.75	557	95.31(2.34,2.35)	59.08
	0.70	–	568	95.14(2.25,2.61)	82.65	565	95.03(2.39,2.58)	82.40
	0.95	–	577	94.56(2.37,3.07)	96.08	574	95.03(2.56,2.41)	96.99
	1.20	–	585	95.12(2.20,2.68)	99.59	581	95.39(2.41,2.20)	99.62
		UQM						
0.05	0.50	–	569	94.77(2.16,3.07)	6.90	566	94.70(2.93,2.37)	6.27
	0.70	–	606	94.97(2.13,2.90)	42.19	602	95.35(2.44,2.21)	41.72
	0.95	–	647	94.62(2.47,2.91)	92.72	643	94.78(2.84,2.38)	92.14
	1.20	–	683	94.75(2.47,2.78)	99.85	679	95.10(2.51,2.39)	99.93
0.10	0.50	–	155	94.14(2.24,3.62)	60.04	152	95.30(2.37,2.33)	58.57
	0.70	–	164	94.56(2.05,3.09)	80.79	160	94.93(2.61,2.46)	81.45
	0.95	–	173	94.70(1.71,3.59)	96.52	169	95.43(2.54,2.03)	96.24
	1.20	–	180	94.07(1.73,4.20)	99.69	177	95.10(2.74,2.16)	99.55
		ICT						
0.05	0.50	–	3243	94.77(2.47,2.76)	7.24	3239	95.00(2.69,2.31)	6.22
	0.70	–	3280	94.86(2.38,2.76)	43.03	3275	94.90(2.36,2.74)	41.02
	0.95	–	3321	94.93(2.43,2.64)	92.88	3316	95.19(2.38,2.43)	92.24
	1.20	–	3357	95.06(2.48,2.46)	99.93	3352	94.59(2.69,2.72)	99.87
0.10	0.50	–	824	94.71(2.54,2.75)	57.42	820	94.85(2.45,2.70)	58.74
	0.70	–	832	94.62(2.34,3.04)	80.84	828	95.27(2.36,2.37)	79.93
	0.95	–	841	94.85(2.26,2.89)	96.09	837	94.52(2.72,2.76)	95.25
	1.20	–	849	95.12(2.40,2.48)	99.71	844	94.78(2.58,2.64)	99.44
		CDM						
0.05	0.60	0.60	500	94.89(2.20,2.91)	0.00	500	94.72(2.66,2.62)	0.00
		0.95	570	94.54(2.68,2.78)	2.65	568	95.07(2.49,2.44)	2.99
		1.10	591	95.02(2.23,2.75)	14.66	589	95.05(2.64,2.31)	15.32
	0.95	0.60	584	94.81(2.42,2.77)	9.16	582	95.07(2.57,2.36)	9.82
		0.95	633	94.96(2.13,2.91)	85.66	631	95.18(2.52,2.30)	87.08
		1.10	647	94.90(2.44,2.66)	97.03	644	95.00(2.53,2.47)	97.40
	1.20	0.60	631	94.95(2.18,2.87)	84.05	629	94.83(2.66,2.51)	84.59
		0.95	667	95.02(2.32,2.66)	99.92	664	94.98(2.48,2.54)	99.98
		1.10	674	94.90(2.20,2.90)	99.99	671	95.01(2.65,2.34)	100.0
0.10	0.60	0.60	137	94.53(2.19,3.28)	8.55	135	95.04(2.78,2.18)	9.98
		0.95	153	94.56(2.06,3.38)	49.53	151	95.10(2.57,2.33)	53.73
		1.10	158	94.12(2.14,3.74)	69.77	155	95.00(2.86,2.14)	69.85
	0.95	0.60	156	94.35(2.17,3.48)	61.50	154	94.97(2.66,2.37)	67.06
		0.95	167	94.99(2.06,2.95)	95.45	164	95.00(2.66,2.34)	96.32
		1.10	169	94.70(2.04,3.26)	97.86	166	94.48(3.07,2.45)	97.75
	1.20	0.60	167	94.92(2.25,2.83)	95.40	164	94.86(2.78,2.36)	95.89
		0.95	174	94.47(2.29,3.24)	99.86	171	95.05(2.66,2.29)	99.91
		1.10	175	94.43(2.25,3.32)	99.92	172	94.84(2.81,2.35)	99.97

$^c$ Expected prevalence ($\pi _\mathrm{es}$) as given by the values of $\pi _s$, i.e., $\pi _\mathrm{es}=(0.50,0.70,0.95,1.20)$ or $(0.60,0.95,1.20) \pi _s$.

$^d$ Expected proportion ($\beta _\mathrm{es}$) as given by the values of $\beta _s$, i.e., $\beta _\mathrm{es}=(0.60,0.95,1.10) \beta _s$.

According to Table 6, when the expected prevalence differs from the true prevalence, we have the following observations: (i) ECPs of all CIs are still very close to the nominal confidence level for each RRT model; (ii) most CIs have satisfactory balance between left- and right-tailed errors; (iii) the closer the expected prevalence rate (i.e., $\pi _{es}$) to the true prevalence rate (i.e., $\pi _s$), the closer the actual assurance probability to the pre-specified assurance probability; (iv) similar observations can be found when the expected cheating parameter differs from the true parameter for the cheater detection model.

## Numerical Examples

To demonstrate the practicability and applicability of the proposed methods, we apply them to the study on unethical behavior in negotiation as discussed in Sect. 1. Suppose that an applied psychologist collects survey data and examines whether negotiators use this unethical tactic to increase the likelihood of reaching an agreement. He or she believes that approximately $16.5\%$ of negotiators make false promises in negotiations (i.e., $\pi _s$ $=$ 0.165). We calculate the required sample size for a new study in which we have $95\%$ chance (i.e., $\beta$ $=$ 0.05) that the half width of the $95\%$ (i.e., $1-\alpha$ $=$ 0.95) confidence interval is no greater than $25\%$ of the point estimate (i.e., $\omega$ = $0.25\pi _s$), for the various RRT models considered in this article.

### Warner’s RRT Model

Within Warner’s RRT model, two mutually exclusive questions about the sensitive attributes are: (A) Have you ever made promises without an intention to deliver in a negotiation? (A$^c$) Have you never made promises without an intention to deliver in a negotiation? These two questions are presented with probability *p* $=$ 0.3 and $1-p$ $=$ 0.7, respectively. With $\pi _s$ $=$ 0.165, the approximate sample size *n* $=$ 3326 is calculated from Eq. (4) based on Wald CI and *n* $=$ 3322 is calculated from Eq. (6) based on Wilson CI, respectively. The corresponding ECPs (EAPs) are $94.93\%$ ($95.89\%$) and $95.03\%$ ($95.73\%$) for Wald and Wilson methods, respectively. In contrast, the conventional sample sizes (i.e., the assurance probability $1-\beta =50\%$) required for a two-sided $95\%$ confidence interval with expected width $\omega$ $=$ $0.25\pi _s$ are *n* $=$ 3274 and 3271, respectively. The corresponding ECPs (EAPs) are $94.87\%$ ($50.95\%$) and $95.15\%$ ($51.16\%$) for the Wald and Wilson methods, respectively.

### Unrelated Question Model

Within the unrelated question model, a neutral question N, for example, “Were you born in odd months?” is required in addition to the sensitive question (A): Have you ever made promises without an intention to deliver in a negotiation? Hence, $\pi _N$ $=$ 0.5. Assume that the sensitive question and the neutral question are presented with probabilities *p* $=$ 0.7 and $1-p$ $=$ 0.3, respectively. With $\pi _s$ $=$ 0.165, the approximate sample size *n* $=$ 950 is calculated from Equation (11) based on Wald CI and *n* $=$ 946 is calculated from Equation (14) based on Wilson CI, respectively. The corresponding ECPs (EAPs) are $94.77\%$ ($96.13\%$) and $94.97\%$ ($95.62\%$) for Wald and Wilson methods, respectively. In contrast, when the conventional sample sizes (i.e., the assurance probability $1-\beta =50\%$) required for a two-sided $95\%$ confidence interval with an expected width $\omega$ $=$ $0.25\pi _s$ are *n* $=$ 898 and 896, the corresponding ECPs (EAPs) are $94.67\%$ ($50.68\%$) and $94.94\%$ ($49.17\%$) for the Wald and Wilson methods, respectively.

### Item Count Technique

Within the model of item count technique, assume that $k=4$ neutral questions are used by a researcher and that the probability of answering each of these neutral question with “yes” is 0.5, i.e., $\pi _i$ $=$ 0.5 for *i* $=$ 1, 2, 3, 4, the number of respondents in control group is the same as that in experiment group, i.e., $n_c$ $=$ $n_e$ $=$ $\frac{1}{2}n$. With $\pi _s$ $=$ 0.165, the approximate sample size *n* $=$ 9756 is calculated from Equation (18) based on Wald CI and *n* $=$ 9748 is calculated from Equation (20) based on Wilson CI, respectively. The corresponding ECPs (EAPs) are $95.04\%$ ($95.83\%$) and $95.05\%$ ($95.56\%$) for Wald and Wilson methods, respectively. In contrast, when the conventional sample sizes (i.e., the assurance probability $1-\beta =50\%$) required for a two-sided $95\%$ confidence interval only with an expected width $\omega$ $=$ $0.25\pi _s$ are *n* $=$ 9652 and 9646, the corresponding ECPs (EAPs) are $94.78\%$ ($49.03\%$) and $95.03\%$ ($50.54\%$) for the Wald and Wilson methods, respectively.

### Cheater Detection Model

Within the cheater detection model, assume that participants in the experiment and control groups receive the sensitive question with probability $p_1$ $=$ 0.2 and $p_2$ $=$ 0.8, respectively. The numbers of participants in two groups are equal, i.e., $n_1$ $=$ $n_2$ $=$ $\frac{1}{2}n$. With $\beta _s$ $=$ 0.7 and $\pi _s$ $=$ 0.165, the approximate sample size of *n* $=$ 1878 is calculated from Equation (26) based on Wald CI and the approximate sample size of *n* $=$ 1872 is calculated from Equation (29) based on Wilson CI, respectively. The corresponding ECPs (EAPs) are $95.04\%$ ($95.92\%$) and $94.78\%$ ($95.95\%$) for the Wald and Wilson methods, respectively. In contrast, when the conventional sample sizes (i.e., the assurance probability $1-\beta =50\%$) required for a two-sided $95\%$ confidence interval with an expected width $\omega$ $=$ $0.25\pi _s$ are *n* $=$ 1800 and 1794, the corresponding ECPs (EAPs) are $94.75\%$ ($49.41\%$) and $95.00\%$ ($49.07\%$) for the Wald and Wilson methods, respectively.

It should be noted that the recommended sample sizes for all models are greater than the number of participants (i.e., 240) recruited to Kern and Chugh’s (2009) studies. In fact, with the sample size 240, the actual ECPs, ECWs and EAPs of CIs for $\pi _s$ under the parameter settings being considered in the above studies for each model are reported in Table 7.

**Table 7:** ECPs(%), ECWs and EAPs(%) of CIs for $\pi _s$ under various RRT models with the actual sample size (i.e.,240 participants) and parameter settings same as those in Sect. 3 for the studies conducted in Kern and Chugh (2009)

	Wald		Wilson	
Model	ECP(ECW)	EAP	ECP(ECW)	EAP
Warner	94.70(0.304)	0.0	94.78(0.302)	0.0
UQM	94.34(0.159)	0.0	95.08(0.158)	0.0
CDM	94.75(0.159)	0.0	95.26(0.158)	0.0
ICT	94.60(0.369)	0.0	95.16(0.366)	0.0

According to the results, the ECPs of CIs for $\pi _s$ under all models are very close to the pre-assigned nominal confidence level (i.e., $95\%$). However, the probabilities of controlling the half width of the CI that are not larger than $\omega =0.25\pi _s=0.04125$ are 0.0 for all models. In fact, the actual half widths of all CIs with sample size 240 are much greater than $\omega =0.25\pi _s=0.04125$, as indicated in Table 7. Specifically, our findings suggest that when the assurance probability is not incorporated into the sample size estimation, the widths of CIs cannot be controlled even though the coverage probability is close to the nominal confidence level.

## Conclusion and Discussion

For studying the prevalence of a sensitive attribute, determining the number of participants to be recruited to the survey is an important research aspect. In the context of survey sampling, sample size determination based on interval estimation is key objective. Therefore, the current research considers sample size determination using interval width control based on two CI types (i.e., Wald and Wilson CIs) for four different randomized response models (i.e., Warner’s model, UQM, ICT and CDM). The derived sample size formulas can control the width of a confidence interval at a specified confidence level with the assurance probability of achieving the pre-specified precision. Our simulation results demonstrate that all formulas derived from Wald and Wilson CIs are accurate in terms of empirical coverage probability (ECP) and empirical assurance probability (EAP). An important note is that sample size formulas based on Wilson CIs outperform those based on Wald CIs for various RRT models in the sense that the ECPs and EAPs of the former are closer to the pre-specified levels than those of the latter. Therefore, sample size formulas derived in this article may help researchers determine a sample size that can achieve pre-specified precision with an assurance probability in a survey study for detecting meaningful prevalence rates. As shown in Ulrich *et al.* (2012), the parameters of the four RRT models cannot be matched with each other; therefore, it is not realistic to compare the sample size formulas of the different RRT models in order to determine the most accurate one. Finally, the numerical examples regarding detecting negotiators’ use of unethical tactics in Sect. 3 clearly demonstrate how to better estimate the required sample size by using interval width control and carrying out a survey instead of conducting a full-scale experiment.

Generally, the two approaches that are employed to determine the desired sample size are, namely hypothesis testing and confidence interval estimation. It is well documented that the former involves both the type I error rate and power, while the latter does not explicitly involve power. In order that the sample size estimation based on expected confidence interval width can provide high assurance in achieving the desired precision, we incorporate an assurance probability for sample size determination to control the width of a confidence interval, i.e., sample size can be estimated by controlling the width of a confidence interval at a specified assurance probability. Ulrich et al. (2012) considered the sample size determination, in terms of hypothesis testing under four randomized response models. However, sample size formulas based on confidence interval width are not available in the extant literature. In this article, we derived such formulas for survey sampling of sensitive attributes. Note that most sample size estimates can be obtained by solving polynomial equation of degree not greater than four. Thus, no complicated computations are required in this article. We also developed program codes to compute the estimated sample sizes, and these program codes are available to readers as the supplementary material.

A restriction of our proposed sample size formulas relies on the specifications of relative accurate values of the prevalence rate (i.e., $\pi _s$) and other model parameters (e.g., $\beta _s$ in CDM model). Researchers, who consider applying our proposed sample size formulas, should be aware that the actual confidence intervals could be wider than required if they cannot come up with accurate approximations of the true prevalence and other model parameters beforehand.

Recently, many variants of the RRT technique have been proposed. For example, Yu, Tian, & Tang (2008) developed a RRT variant (i.e., the crosswise model (CWM) with simple instructions. Specifically, the interviewee received a sensitive question (denoted as "S") and a neutral question (denoted as "N") simultaneously and was then asked to indicate whether the two answers given in "S" and "N" were the same or different. This model can be applied to both face-to-face personal interviews and mail questionnaire, because no randomization device is required. This also enables the interviewee to mask his or her answer to the sensitive question. Therefore, it has received considerable attention in the scientific community (e.g., Sagoe et al., 2021; Schnell & Thomas, 2021). It is clear that the crosswise model is mathematically equivalent as Warner’s model. As a result, the reported formulas and the results of the Warner model are equally valid for CWM. Ostapczuk et al. (2009) proposed a symmetric variant of CDM, but an iterative algorithm (e.g., expectation-maximization (EM) algorithm (Dempster, Laird, & Rubin, 1977)) is needed to obtain the maximum likelihood estimations (MLEs) of parameters $\pi _s$, $\beta _s$ and $\gamma$. Therefore, we do not consider the sample size determination under the symmetric CDM in this article.
